# Synaptogyrin-2 influences replication of Porcine circovirus 2

**DOI:** 10.1371/journal.pgen.1007750

**Published:** 2018-10-31

**Authors:** Lianna R. Walker, Taylor B. Engle, Hiep Vu, Emily R. Tosky, Dan J. Nonneman, Timothy P. L. Smith, Tudor Borza, Thomas E. Burkey, Graham S. Plastow, Stephen D. Kachman, Daniel C. Ciobanu

**Affiliations:** 1 Animal Science Department, University of Nebraska, Lincoln, Nebraska, United States of America; 2 USDA, ARS, U.S. Meat Animal Research Center, Clay Center, Nebraska, United States of America; 3 Department of Plant, Food and Environmental Sciences, Faculty of Agriculture, Dalhousie University, Truro, Canada; 4 Department of Agricultural, Food and Nutritional Science, University of Alberta, Edmonton, Canada; 5 Department of Statistics, University of Nebraska, Lincoln, Nebraska, United States of America; University of Bern, SWITZERLAND

## Abstract

Porcine circovirus 2 (PCV2) is a circular single-stranded DNA virus responsible for a group of diseases collectively known as PCV2 Associated Diseases (PCVAD). Variation in the incidence and severity of PCVAD exists between pigs suggesting a host genetic component involved in pathogenesis. A large-scale genome-wide association study of experimentally infected pigs (n = 974), provided evidence of a host genetic role in PCV2 viremia, immune response and growth during challenge. Host genotype explained 64% of the phenotypic variation for overall viral load, with two major Quantitative Trait Loci (QTL) identified on chromosome 7 (SSC7) near the swine leukocyte antigen complex class II locus and on the proximal end of chromosome 12 (SSC12). The SNP having the strongest association, *ALGA0110477* (SSC12), explained 9.3% of the genetic and 6.2% of the phenotypic variance for viral load. Dissection of the SSC12 QTL based on gene annotation, genomic and RNA-sequencing, suggested that a missense mutation in the *SYNGR2* (*SYNGR2 p*.*Arg63Cys*) gene is potentially responsible for the variation in viremia. This polymorphism, located within a protein domain conserved across mammals, results in an amino acid variant *SYNGR2 p*.*63Cys* only observed in swine. PCV2 titer in PK15 cells decreased when the expression of *SYNGR2* was silenced by specific-siRNA, indicating a role of *SYNGR2* in viral replication. Additionally, a PK15 edited clone generated by CRISPR-Cas9, carrying a partial deletion of the second exon that harbors a key domain and the *SYNGR2 p*.*Arg63Cys*, was associated with a lower viral titer compared to wildtype PK15 cells (>24 hpi) and supernatant (>48hpi)(P < 0.05). Identification of a non-conservative substitution in this key domain of *SYNGR2* suggests that the *SYNGR2 p*.*Arg63Cys* variant may underlie the observed genetic effect on viral load.

## Author summary

The cost of managing Porcine Circovirus 2 (PCV2) associated diseases in the US alone costs the swine industry more than $250 million a year. This virus is found in all swine populations in the US, but only a few pigs get sick and show signs of disease. Previous anecdotal field data showed differences between pig breeds in both incidence and severity of PCV2-associated diseases, supporting the role of host genetics in disease susceptibility. This research, including over 1,000 experimentally infected pigs with PCV2, is the largest study ever conducted to understand the role of host genetics in PCV2 related illnesses. We found that a pig’s own genetics can impact the ability of PCV2 to multiply and cause disease. Specifically, we found a mutation in the *SYNGR2* gene that influences the ability of the PCV2 virus to replicate, which can affect pig growth and immune system following infection. This research will aid in the development of genetic tests with the ability to predict PCV2 susceptibility. Early identification of pigs that are susceptible to PCV2 infection will result in an improvement in the general health and welfare of pigs.

## Introduction

Porcine Circovirus 2 (PCV2) is a member of the *Circoviridae* family and the smallest virus known to infect mammalian cells. Despite its small size, this single-stranded circular DNA virus has been identified as the causative source of a set of systemic disorders known as Porcine Circovirus Associated Diseases (PCVAD), which includes Post-Weaning Multi-systemic Wasting Syndrome (PMWS). PMWS is characterized by severe weight loss, respiratory and enteritic conditions that can lead to mortality [1]. Other symptoms associated with PCVAD include nephritis, dermatitis, reproductive failure, interstitial pneumonia, and lymphoid depletion. PCV2 infection can be detected in all domestic populations of pigs, but most infections are subclinical and only a subset of pigs that experience various triggering factors develop clinical disease [2]. The frequency of subclinical infection, combined with environmental stability of the virus, has enabled PCV2 to spread worldwide and persist undetected for generations. For example, the first documented PMWS outbreak occurred in 1991, but PCV2 was identified in archival semen samples collected in the early 1970s [3]. Current PCV2 isolates display consistent variation in a 9 bp region of the capsid gene, associated with increased virulence in experimental infection of gnotobiotic pigs, compared to archival PCV2 isolates, indicating viral genetic variation associated with virulence [3].

Anecdotal field data and initial experimental evidence [4, 5] described differences between breeds in both incidence and severity of PCVAD [6], supporting the role for host genetic variation in the etiology of the disease. In our first genome-wide association study (GWAS), we found that host genetics influenced PCV2 titer and accounted for an important proportion of the phenotypic variation (~45%) for viral load [7]. In this study, we integrated two datasets of pigs experimentally infected with a PCV2b strain [7, 8] and *in vitro* siRNA and gene editing validation models to elucidate the role of host genetics in pathogenesis by identifying genes and genetic variants that could influence PCV2 susceptibility.

## Results

### Two genomic loci influence host genetic effect on PCV2 infection

Substantial variation in the timing and magnitude of immune response, and in the efficiency of PCV2 replication, was reported in our previous studies of experimental infection with a PCV2b strain [7, 8]. The present study extends that work by examining the influence of host genetics on the process of infection, based on a combination of the two previous study populations (n = 974 F1 crossbred pigs originating from 14 genetic lines) challenged by experimental infection with PCV2b and genotyped with 56,557 SNPs (Porcine SNP60 BeadArray). The population structure provides substantial variation in linkage disequilibrium (LD) decay in order to identify genomic regions that influence the phenotype using a GWAS approach. Key phenotypes included viremia and PCV2-specific antibodies at specific time points, overall viral load across the study, and growth rate as a measure of impact of infection. The proportion of phenotypic variation accounted for by SNP genotypes was limited early in the infection, but increased after the surge in viral replication and associated immune response. Specifically, SNP genotypes explained from 19% of phenotypic variation in PCV2 viremia at 7 days post infection (7 dpi) to 52% at 14 dpi (Table 1). Similarly, SNP genotypes explained 14% and 3% of PCV2-specific IgM and IgG variation at 7 dpi, respectively, but this increased to 60% (IgM) and 44% (IgG) at 21 dpi. Overall, SNP genotypes explained 64% of the variation in viral load calculated across time points. In comparison, the contribution of SNP genotypes to variation in Average Daily Gain (ADG, monitoring growth rate through body weight) during the study period was limited, explaining 16% of the variation in overall ADG with 13% explained at 7 dpi and 7% at both 21 and 28 dpi.

**Table 1:** Proportion of phenotypic variance explained by SNPs from Porcine SNP60 BeadArray using BayesB of PCV2-related traits days post infection (dpi) with PCV2.

Trait/dpi	7	14	21	28	0–28
Viremia	0.19	0.52	0.45	0.39	0.64
IgM	0.14	0.60	0.44	0.52	
IgG	0.03	0.08	0.44	0.38	
	0–7	7–14	14–21	21–28	0–28
ADG	0.13	0.11	0.07	0.07	0.16

**Fig 1:**
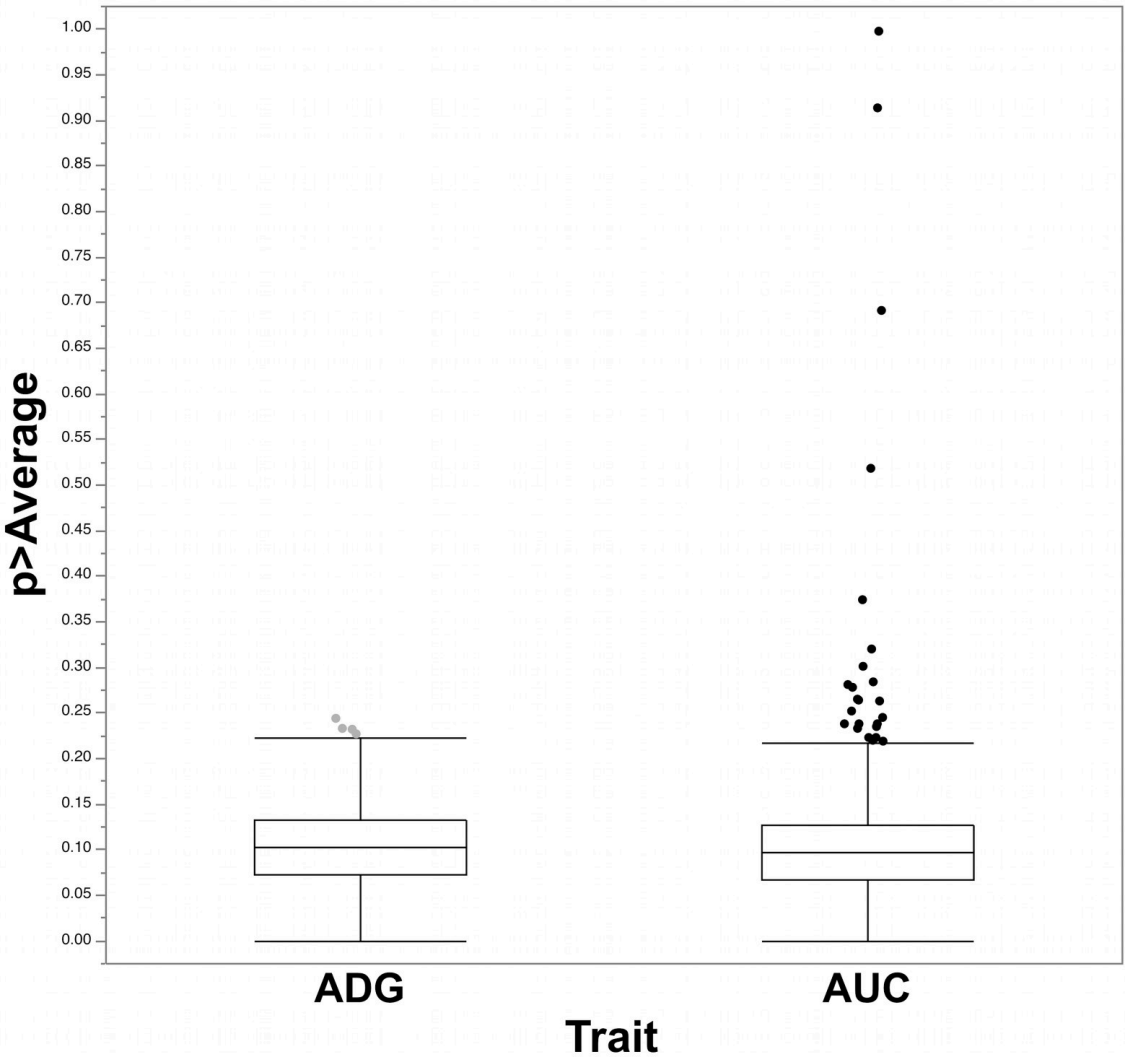
Probability of a 1 Mb window to have an effect above the average effect of the genome-wide 1 Mb windows estimated using BayesB on overall average daily gain (ADG) and PCV2b viral load (AUC). The genetic variance for PCV2 viral load explained by the top two 1 Mb windows that included *ALGA0039682* (SSC7) and *ALGA0110477* (SSC12) have effects above the 1 Mb window average effect (P > 0.90).

The initial GWAS was performed using a BayesB-based approach where individual SNPs and successive 1 Mb windows of the genome were evaluated for association with phenotypic variation [7]. Bayesian regression models fit multiple SNPs in genome-wide associations, assuming that the marker effects result from a mixture of a point mass distribution whereby SNP have null effects and a distribution of non-zero effects (e.g., normal, heavy tailed). Prior assumptions are made relative to the genetic and environmental variances and the proportion of markers that have a null effect on a specific trait of interest. These models are implemented via a Markov chain Monte Carlo (MCMC) sampling algorithm. The posterior means are averaged over the number of samples from the MCMC [9]. Genome-wide average posterior distribution for the genetic variance was used to estimate the probability of each 1 Mb window having greater than the average genetic variance explained across PCV2-related traits (S1 Table). The analysis identified two windows with greater than average window effect associated with both viral replication and immune response phenotypes (Pr > 0.90), assumed to represent Quantitative Trait Loci (QTL) (Fig 1). One window associated with viral load was found on SSC7 in the vicinity of the swine leukocyte antigen complex class II (*SLAII*) at 24–25 Mb while the other was located near the proximal end of SSC12, at 3–4 Mb. The SNPs associated with the largest genetic variance, *ALGA0039682* and *ALGA0110477*, in each of these two windows explained 65.1% and 99.7%, respectively of the genetic variance explained by their respective windows.

The SNP (*ALGA0110477*) associated with the largest effect on viral load explained 9.3% of the genetic variance and 6.2% of the phenotypic variance for PCV2 viral load (S1 Fig). This SNP was initially located on an unplaced scaffold in the previous 10.2 reference assembly. Estimating LD between *ALGA0110477* and all other SNPs on the Porcine SNP60 BeadArray provided weak evidence of its location at the proximal end of the SSC12 reference sequence. Specifically, SNPs *ALGA0122316*, *ASGA0089708*, and *ASGA0090188* had the highest LD estimates with *ALGA0110477* (r^2^ = 0.28–0.33). Interestingly, SNPs in the genomic region encompassing these markers did not show strong evidence of association with viral load despite LD with *ALGA0110477*. A more nuanced analysis fitting haplotypes across the region rather than individual SNPs using BayesIM [10] detected an effect in this genomic region (S2 Fig), without the inclusion of the previously unmapped *ALGA0110477*, providing support for the initial discovery based only on *ALGA0110477*. The unplaced scaffold containing the SSC12 marker, *ALGA0110477*, did not contain any annotated candidate genes that might underlie the observed effects, and the available sequence surrounding the marker only extended for 84 bp. Using inverse PCR (iPCR), the proximal sequence was extended to 1,252 bp. This extended sequence was used to interrogate contigs from an early version of a long read-based genome assembly of a pig (accession NPJO00000000), [11] which identified a 19 Mb scaffold that provided precise location and context for *ALGA0110477* used for identification of candidates genes described below. The recent release of a long read-based improved reference assembly, *Sscrofa* 11.1 (GenBank accession GCA_000003025), supported more accurate ordering and placement of markers, including *ALGA0110477* (SSC12, 3,673,576 bp).

**Fig 2:**
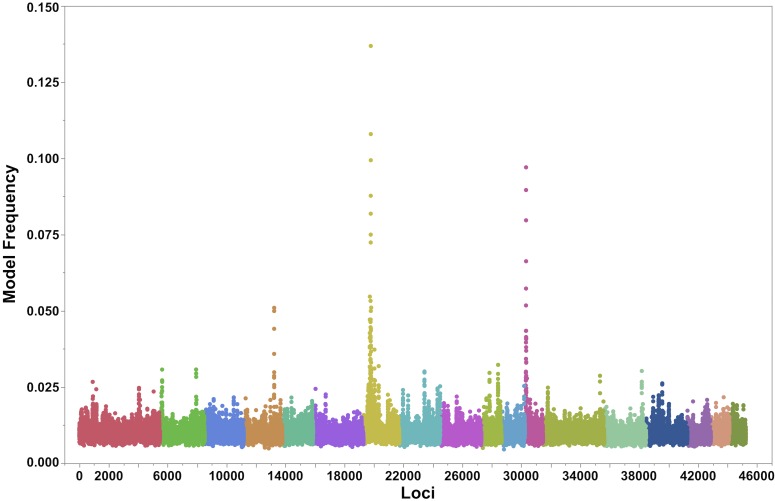
Genome-wide association between 51,592 SNPs and PCV2b viral load using BayesIM. Each dot represents the model frequency associated with each 50kb QTL. The X-axis represents the position of the 50 kb loci across the swine genome using *Sscrofa* 11.1 assembly. The Y-axis represents the model frequency of the association between a QTL and PCV2b viral load. Alternate colors represent autosomes, from SSC1 to 18.

Profiling of the loci associated with PCV2-related phenotypes across time points following infection was performed in order to distinguish the role of host genetics in innate and adaptive immunity. One of the outputs of Bayesian analyses is model frequency, which provides the proportion of post-burn-in samplings that included a particular SNP covariate in the model. Model frequency can also be used to compare loci effects across multiple traits, despite differences in phenotypic and genetic variances. This analysis based on BayesIM supported the previous result, with the highest model frequency for viral load occurring within the previously identified locations on SSC7 and SSC12 (Fig 2). In addition, haplotype effects estimated across the proximal end of SSC12 (0–10 Mb), provided evidence of haplotypes with divergent effects in viral load, with a peak detected at 3.7 Mb (S3 Fig). The two major QTLs were consistently observed for other targeted traits, including viremia (S4 Fig). Both SSC7 and SSC12 QTLs had similar model frequencies at 14 dpi, while the QTL on SSC7 showed an increasing effect on viremia at 21 and 28 dpi. It should be noted that an additional QTL on the proximal end of SSC8 was detected for viremia at 14 dpi; this QTL had not been observed for viral load, and represented the largest effect for 14 dpi. The QTLs located on SSC7 and SSC12 were also observed for PCV2-specific antibody variation. Specifically, these QTLs were associated with IgM variation, indicative of active infection, starting at 14 dpi and with IgG variation, representing previous PCV2 exposure or vaccination, starting at 21 dpi (S5 and S6 Figs), supporting the hypothesis that they represent host variation affecting PCV2 infection including immune response.

### SLAII role in PCV2 replication and immune response

One of the genomic regions associated with viral load was located on SSC7, in the vicinity of *SLAII* locus. The SNPs associated with the largest effects (*ALGA0039682* and *ALGA0039710*, at 24.5 and 24.8 Mb, respectively) are located at the proximal end of *SLAII* with *DRA* being the closest gene (24.8 Mb) from the *SLAII* complex. Combined, these two SNPs explained 3.8% of the genetic variance for viral load. While the role of *SLAII* in antigen recognition and immune response in a variety of infectious diseases is well established, highly polymorphic genes and extended LD are the main factors limiting the discovery of functional variants. The SNP *ALGA0039710*, was still associated with the largest effect in viral load in an analysis of a subset of samples (n = 268) with extreme phenotypes that included novel SNPs located in genes in the QTL such as *DRA*, *C2*, *CFB*, *NELFE*, *SKIV2L* [12].

### Fine mapping and annotation of the QTL region located at the proximal end of SSC12

Previous to the recent release of the *Sscrofa* 11.1, we used the un-annotated long-read scaffold to identify the genes surrounding the SSC12 marker *ALGA0110477*. *Ab initio* gene prediction [13] and pBLAST combined with RNA-seq of peripheral blood were used for annotation of this QTL region. Thirteen potential genes with an e value > 7e^-64^ and a pBLAST score > 200 were identified. Five of these genes were found to be expressed in RNA-seq data of peripheral blood from pigs subjected to PCV2. These genes are involved in immune response and cytokine signaling (*SOCS3*), inhibition of apoptosis and promotion of cell proliferation (*BIRC5*), membrane trafficking and transport (*SYNGR2*) and transmembrane ion channels (*TMC6* and *TMC8*). The number of isoforms observed across these genes varied from one (*SOCS3*) to more than 10 (*TMC6*).

**Fig 3:**
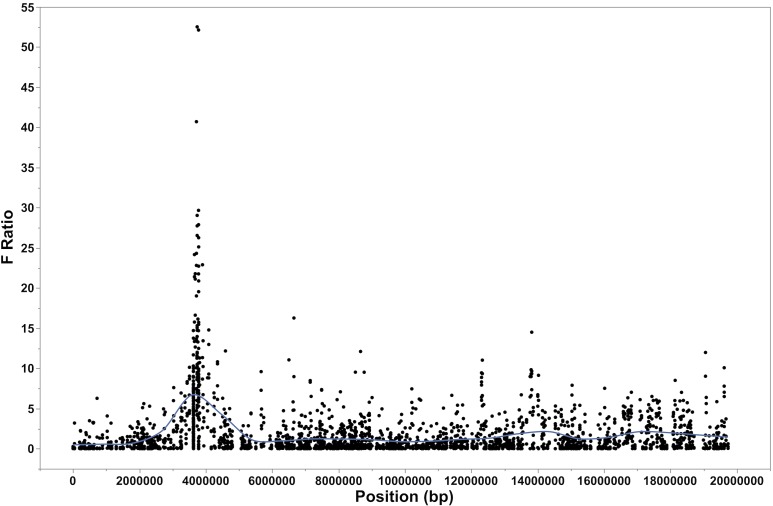
Association results between the genotypes of the DNA polymorphisms mapped to the 19 Mb scaffold of the proximal end of SSC12 and PCV2b viral load using a linear mixed additive model. The line represents a smoother with a default λ of 0.05. *SYNGR2 p*.*Arg63Cys* and *BIRC5 g*.*-343delA* were associated with the largest effects on PCV2b viral load (F-ratio > 47, *P* < 0.0001).

RNA-seq analysis of alternate *ALGA0110477* homozygotes exhibiting extreme viral load following PCV2 challenge uncovered missense (n = 4), synonymous (n = 11), and UTR (n = 10) SNPs and an UTR indel across the 5 candidate genes located in the QTL region. In addition, 1–2 kb sequencing upstream of the Transcription Start Site (TSS) for *BIRC5*, *SOCS3* and *SYNGR2* uncovered 32 SNPs and 4 short indels. These novel polymorphisms and 580 SNPs from the Porcine SNP60 BeadArray were mapped to the 19 Mb scaffold using BLAT. The highest LD between *ALGA0110477* and the polymorphisms mapped on the scaffold was with a SNP from the Porcine SNP60 BeadArray (*ASGA0086395*, r^2^ = 0.55) located 24.5 kb away followed by a group of 3 SNPs from *SYNGR2* (r^2^ = 0.42–0.48) including the missense polymorphism *SYNGR2 p*.*Arg63Cys* located 123.7 kb away. Using an additive linear mixed model and a subset of pigs with extreme high and low viral loads (n = 268) genotyped for all polymorphisms mapped to the scaffold (n = 629), we found that the *SYNGR2 p*.*Arg63Cys* SNP and a 1bp indel located 343 bp upstream of *BIRC5* TSS were associated with the largest effects on PCV2 viral load (Fig 3, F-ratio > 47, *P* < 0.0001). The phenotypic variance explained by each of these novel polymorphisms was substantially larger (21–23% +/- 6.1–6.4%) compared to the original QTL SNP *ALGA0110477* (12.6 +/- 4.8%). As expected, these polymorphisms were associated with large effects on all weekly viremia measures (*P < 0*.*0001*), and on PCV2-specific antibodies, starting from 14 dpi for IgM and 21 dpi for IgG (p < 0.0001). The effects on growth during the challenge were most evident after 14 dpi as well as during the entire challenge period (0–28 dpi, P < 0.0005).

### *SYNGR2* a potential candidate gene influencing PCV2 replication

**Fig 4:**
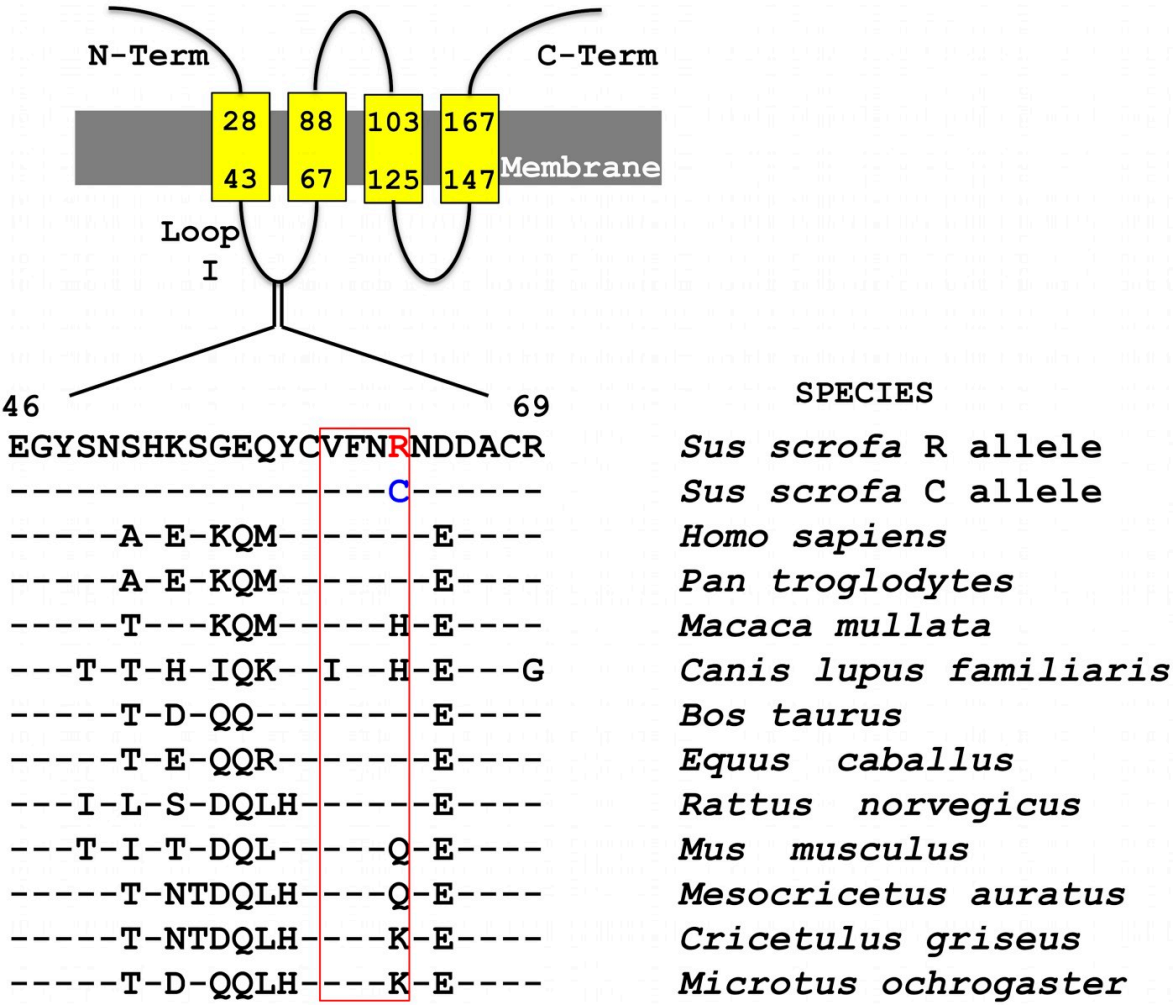
Sequence alignment of the first loop of SYNGR2 across mammalian species. The red block indicates a conserved region demonstrated to be important in successful incorporation of the protein into vesicular membranes and vesicle formation.

**Fig 5:**
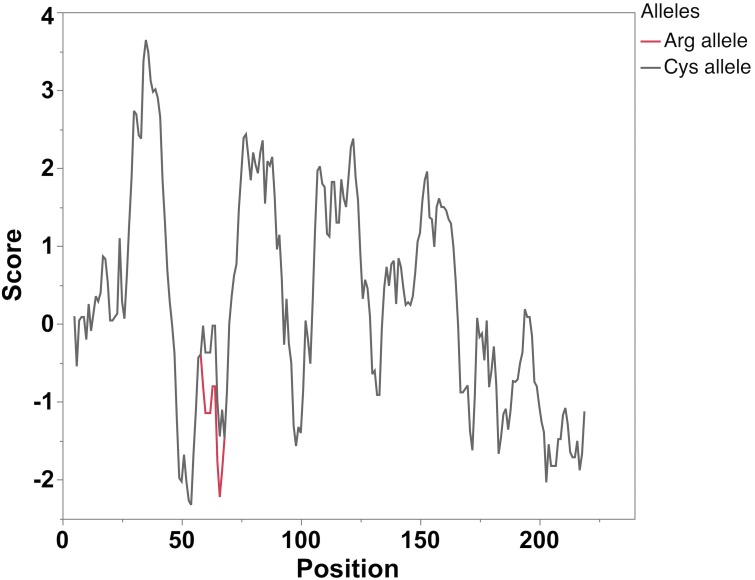
Hydrophobicity profile of the *SYNGR2 Arg63Cys* polypeptides based on the Kyte and Doolittle scale (the window consisted of 9 amino acid residues). An increase in the hydrophobicity score was observed in the predicted *SYNGR2 p*.*63Cys* polypeptide.

The *SYNGR2 p*.*Arg63Cys* SNP (SSC12, 3,797,516 bp) is located in the first loop of synaptogyrin-2 (SYNGR2) in a region conserved across mammals [14] known to be crucial for formation of microvesicle membrane fraction [15]. The *Arg* residue is prevalent in other species (e.g., human, rat, cow, horse) sometimes being replaced by *His* (Rhesus macaque, dog), *Lys* (prairie vole, Chinese hamster) or *Gln* (mouse, golden hamster) while the *Cys* residue appears to be specific to swine (Fig 4). The substitution of *Arg* to *Cys* determines a change in charge and hydrophobicity of the loop (Fig 5).

**Fig 6:**
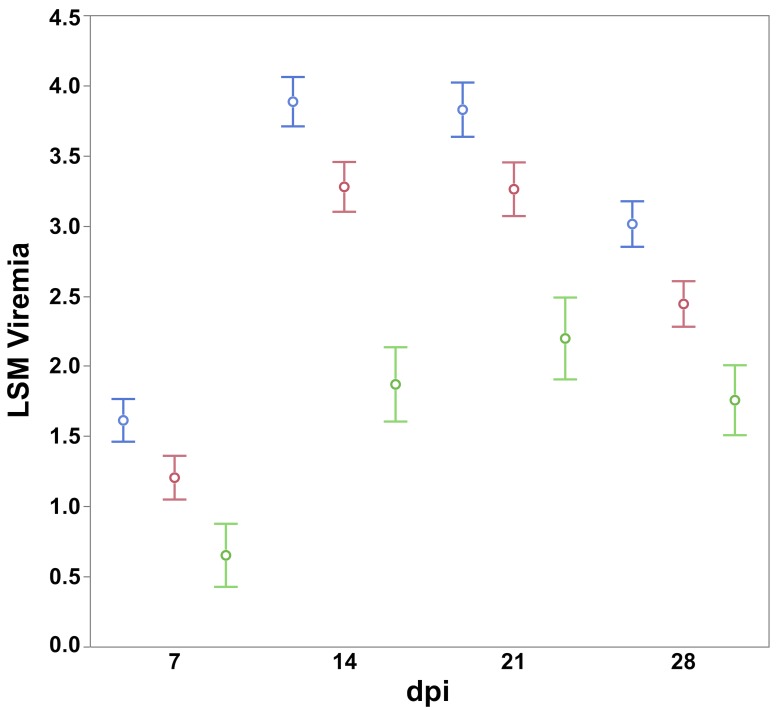
Least square means and standard errors of the *SYNGR2 p*.*Arg63Cys* genotypes (*63Cys /63Cys*—green, *63Arg /63Cys—*red, *63Arg/63Arg—*blue) across weekly viremia measures following PCV2b challenge (n = 268).

The *SYNGR2 p*.*63Cys* allele is favorable with the viral load of the homozygous genotype (Least Square Mean = 54.3 units) being lower compared to the heterozygote (67.03 units, *P = 0*.*005*) and alternate homozygote (*p*.*63Arg*, 79.54 units, *P < 0*.*0001*). The favorable homozygous genotype was also associated with lower weekly viremia (P < 0.0001, Fig 6), IgM (> 14 dpi, P < 0.0001, S7 Fig), IgG (> 21 dpi, P < 0.0001, S8 Fig) and higher growth (overall 0–28 dpi and > 14 dpi, P < 0.001) compared to the alternate homozygote. We hypothesize that the effects on growth and PCV2 specific antibodies are a result of the variation in viremia modulated by *SYNGR2*. Expression of *SYNGR2* did not differ across *SYNGR2 p*.*Arg63Cys* genotypes or time points following *in vivo* PCV2 challenge. No interaction (P > 0.30) was detected between *SYNGR2 p*.*Arg63Cys* and the SNPs associated with the largest effects from the QTL detected on SSC7 (*ALGA0039682* and *ALGA0039710*). The effect of this SNP was confirmed in an independent validation data set consisting of 71 pigs infected with the same PCV2b strain and representing all three *SYNGR2 p*.*Arg63Cys* genotypes. This SNP had an effect on viremia starting from 14 dpi to 42 dpi (P < 0.05). The viremia of the homozygous genotype for *SYNGR2 p*.*63Cys* allele was lower than the alternate homozygote (14–28 dpi, P < 0.05) and the heterozygote (14 dpi, P < 0.05).

Our inability to uncover the QTL located on SSC12 in our previous report [7] was based on 1) a genetic structure with a very limited number of homozygotes for *SYNGR2 p*.*63Cys* allele (Q = 1.2%) compared to Engle et al. (2014) dataset (Q = 18.3%), which is less favorable for detecting associations in additive statistical models, and 2) lower ability of the *ALGA0110477* to capture the low viremic effects of *SYNGR2 p*.*63Cys*. While in the Engle et al. (2014) dataset the presence of the *ALGA0110477 C* variant had a probability of 65% to be located on the same haplotype with *SYNGR2 p*.*63Cys*, in McKnite et al. (2014) this variant is found in similar proportions in haplotypes that carry different *SYNGR2* alleles (e.g. *SYNGR2 p*.*63Cys*; P = 55.9%).

The 1bp deletion located 343 bp upstream of the TSS of *BIRC5* (*BIRC5 g*.*-343delA*) was found to be in high LD (r^2^ = 0.83) with *SYNGR2 p*.*63Cys* allele and as expected was associated with low viral load (P < 0.0001). The deletion was predicted to affect a potential motif for *NR5A2*, a DNA-binding zinc finger transcription factor. However, no significant difference in expression was observed between *BIRC5* genotypes across time points following *in vivo* PCV2 challenge (P < 0.17). At 14 dpi the homozygotes for the insertion exhibited an elevated nominal expression compared to the other genotypes, but the difference was not significant (P = 0.061).

Located 41.9 kb apart, the high LD observed between *SYNGR2 p*.*Arg63Cys* and *BIRC5 g*.*-343delA* (r^2^ = 0.83) hampered the ability to distinguish their individual effects in the *in vivo* challenge dataset. In contrast, the LD between *SYNGR2 p*.*Arg63Cys* and other *SYNGR2* SNPs was limited (r^2^ < 0.26), as well as the LD between *BIRC5 g*.*-343delA* and other *BIRC5* polymorphisms (r^2^ < 0.16). A very defined LD block exists from *ALGA0110477* to *SYNGR2* that includes 16 DNA polymorphisms. Within this block, there were 9 haplotypes with individual frequencies greater than 1% that accounted for 85% of the haplotypes present. A single haplotype (*Hap 1*) carried the *SYNGR2 p*.*63Cys* allele. The frequency of this haplotype in our resource population was 0.28. The remaining eight haplotypes carried the *SYNGR2 p*.*63Arg* allele. A haplotype substitution effect demonstrates that *Hap 1* was associated with the lowest viral load (P < 0.0001, S2 Table) substantiating the potential role of *SYNGR2 p*.*Arg63Cys* in PCV2 susceptibility.

**Fig 7:**
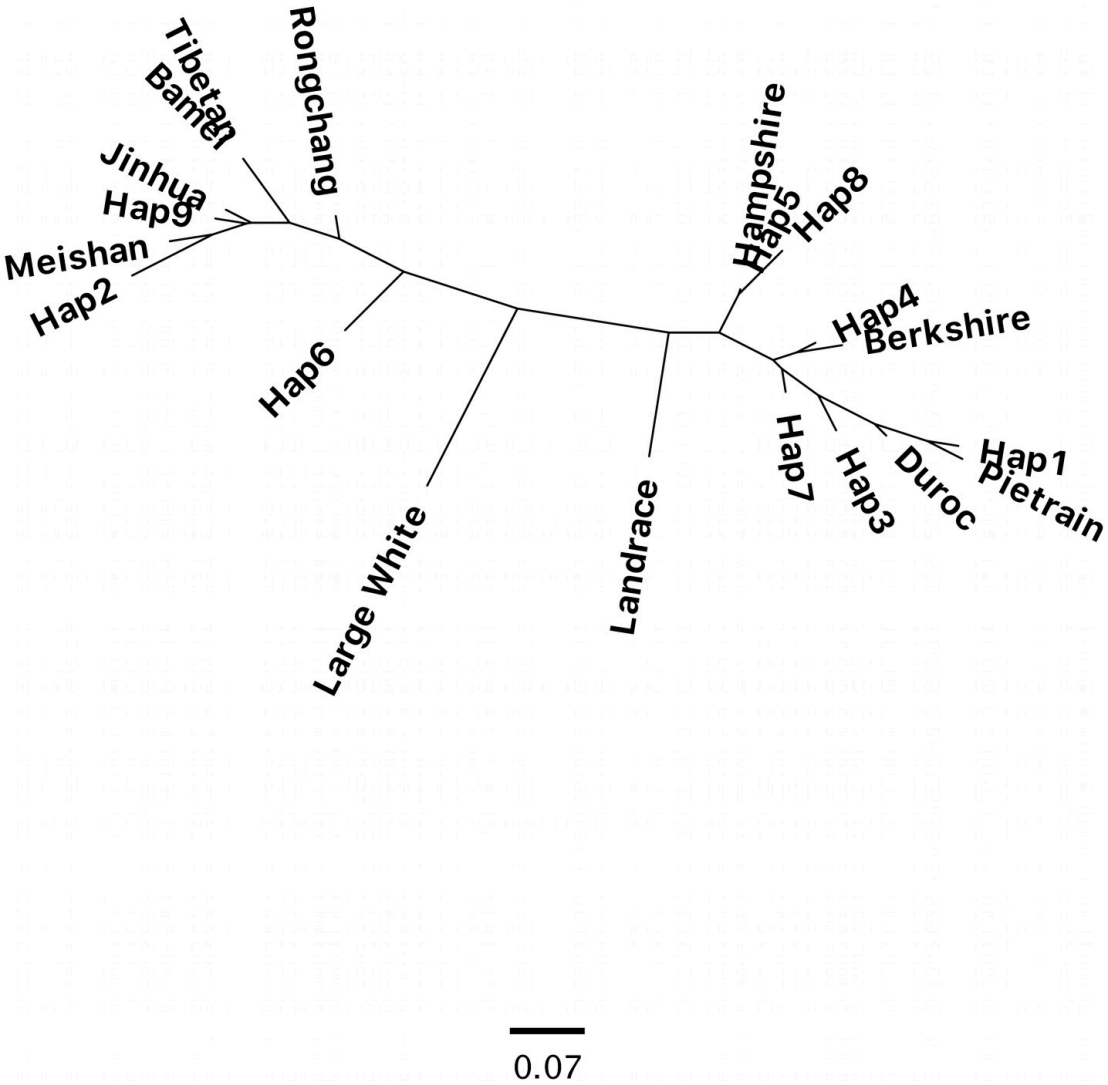
Phylogenetic tree of haplotypes from the LD block between *ALGA0110477* and *SYNGR2* (SSC12) in our resource population (*Hap1–9*) and selected domestic pig breeds.

An analysis of the Sequence Read Archive and Whole-genome Shotgun Sequences revealed that the *SYNGR2 p*.*63Cys* allele is only present in Duroc and Pietrain, while *SYNGR2 p*.*63Arg* was present in the rest of the Western (Large White, Landrace, Hampshire and Berkshire) and indigenous Chinese breeds (Bamei, Jinhua, Meishan, Rongchang and Tibetan), as well as wild pigs such as common Warthog (*Phacochoerus africanus*), Java (*Sus verrucosus*) and Visayan (*Sus cebifrons*) warty pigs. Phylogenetic analysis based on the polymorphisms from this haplotype block separated the breeds into paternal (Pietrain, Duroc, Hampshire and Duroc) and indigenous Asian breeds while the maternal breeds (Large White and Landrace) were located in between these two groups (Fig 7). Geographical location has an important role in the genetic relationships between indigenous Chinese breeds [16]. The breeds originating from the Tibetan plateau (Tibetan pigs from Sichuan region and Bamei pigs) share the same haplotype, which is similar with the haplotype of the pigs from the neighboring Rongchang region. In contrast, Meishan and Jinhua are from the middle-lower belt of Yangtze River and their haplotypes share more similarities.

The haplotype that carried the *SYNGR2 p*.*63Cys* allele (*Hap1*) was the closest to Pietrain and Duroc. The other haplotypes present in our resource population were similar to those identified in Berkshire (*Hap 4*), Hampshire (*Hap 5*), Meishan (*Hap 2*), or Jinhua (*Hap 9*). This finding suggests that *SYNGR2 p*.*63Cys* is the predominant allele in Duroc and Pietrain, breeds in which more emphasis is placed on growth related traits compared to breeds such as Large White and Yorkshire. Our analysis of *SYNGR2 p*.*Arg63Cys* in pure breeds showed that *SYNGR2 p*.*63Cys* has a frequency of 0.25 in Yorkshire, 0.53 in Landrace and 0.78 in Duroc.

### Viral titer is decreased in *SYNGR2*-silenced cells infected with PCV2

**Fig 8:**
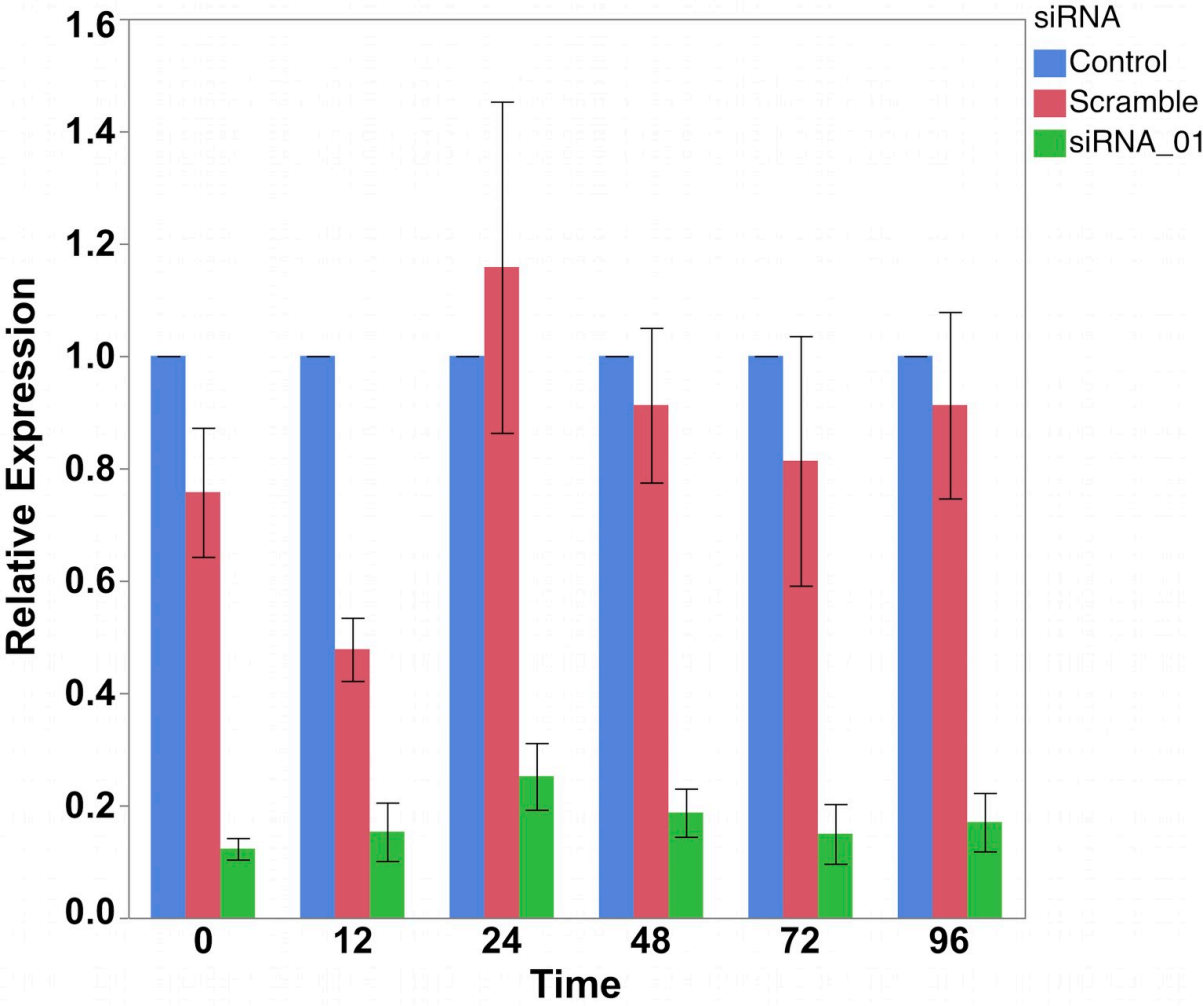
Expression of *SYNGR2* in PK15 cells transfected with *SYNGR2* specific siRNA-01, scramble siRNA and non-transfected controls following inoculation with the UNL2014001 PCV2b strain. Expression of *SYNGR2* in siRNA-01 and scramble siRNA treated cells is presented as relative expression to the control cells.

**Fig 9:**
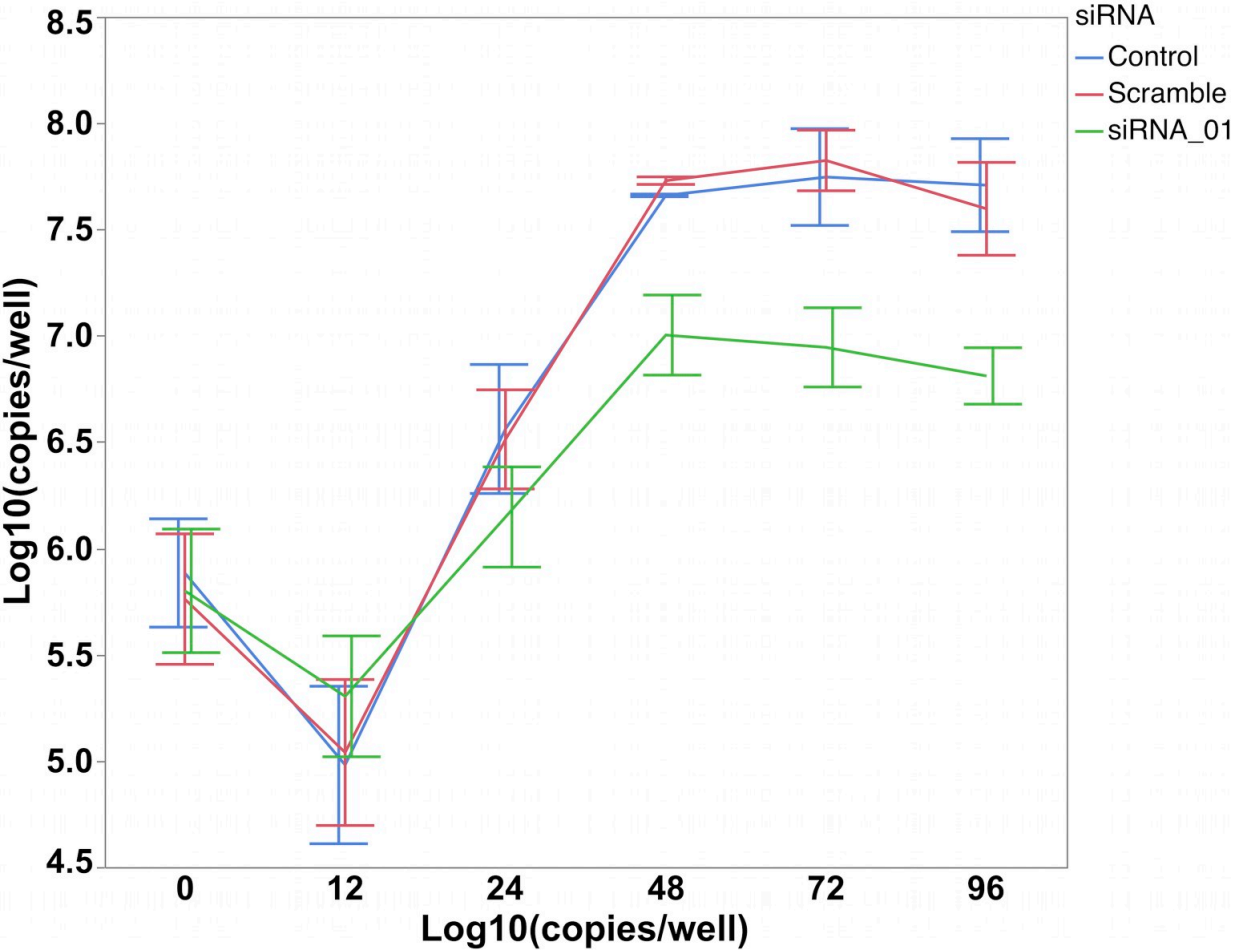
PCV2 copy number in PK15 cells transfected with *SYNGR2* specific siRNA-01, scramble siRNA and non-transfected controls following inoculation with the UNL2014001 PCV2b strain. The number of viral copies from PK15 cells is expressed as log10 copies/well.

Considering that the location of the *SYNGR2 p*.*Arg63Cys* substitution is in a conserved domain involved in vesicle formation [15] and recent literature support of *SYNGR2* affecting replication of a tick-borne human RNA virus [17], we hypothesized that *SYNGR2* may play a role in the internalization and early release of PCV2 from endosomes influencing its replication. The Porcine kidney 15 cell line (PK15) has an epithelial origin and is a well-established model system for PCV2 innate immunity and cellular pathogenesis [2]. We found that PK15 cells carry both the *SYNGR2 p*.*63Arg* and the insertion of *BIRC5 g*.*-343delA* variants associated with high-viremia. Expression of *SYNGR2* did not differ across time points following PCV2 infection of PK15 cells, corroborating *in vivo* findings. In order to validate a role of *SYNGR2* in PCV2 replication, we transfected PK15 with siRNA targeting the mRNA of *SYNGR2*. We evaluated two siRNA (siRNA-01 and siRNA-03) at two different concentrations (10 nM and 20 nM) and found that siRNA-01 was the most efficient to knock-down mRNA level of *SYNGR2* compared to the cells subjected to a scramble siRNA control. A substantial reduction (>75%) in *SYNGR2* mRNA level was observed starting 24 hours after transfection (Fig 8). PK15 cells with the expression of *SYNGR2* silenced were then infected with PCV2 24 hours after transfection. A reduction in viral titer was observed in the *SYNGR2* silenced cells subjected to PCV2b starting at 48 hours post infection (hpi) when compared to scramble siRNA and non-transfected control cells, indicating a role of *SYNGR2* in viral replication (Fig 9) (P < 0.05). The viral titer across time points was not statistically different between the scramble siRNA and non-transfected control cells (P > 0.54).

### PCV2b titer is significantly lower in PK15 cells that carry an edited SYNGR2

**Fig 10:**
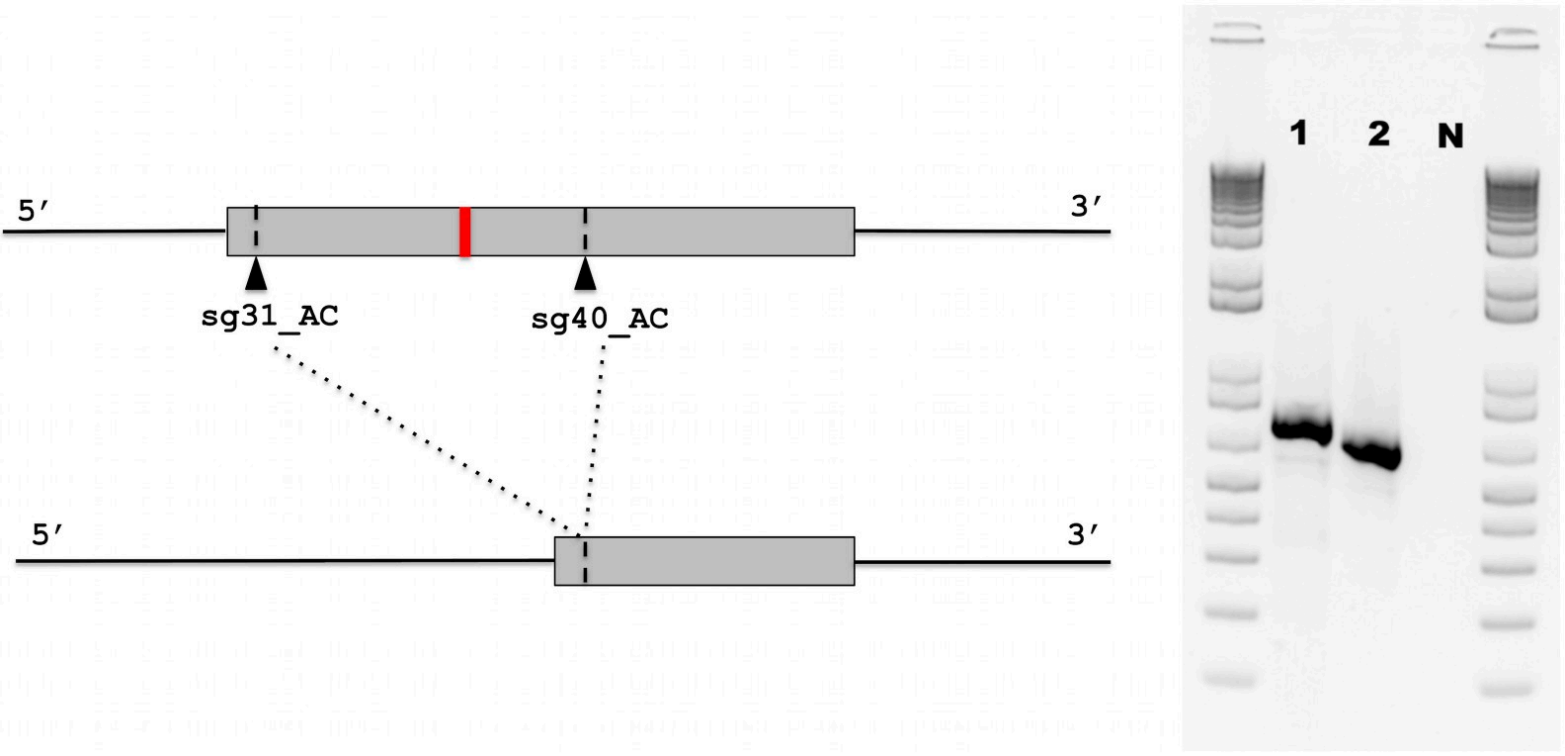
CRISPR Cas9 guide RNA design. Position of selected guide RNA in the second exon of *SYNGR2* relative to the *SYNGR2 p*.*Arg63Cys* polymorphism resulting in a 106 bp deletion in the E1 edited clone (2) compared to wildtype PK15 (1) observed by agarose gel electrophoresis.

**Fig 11:**
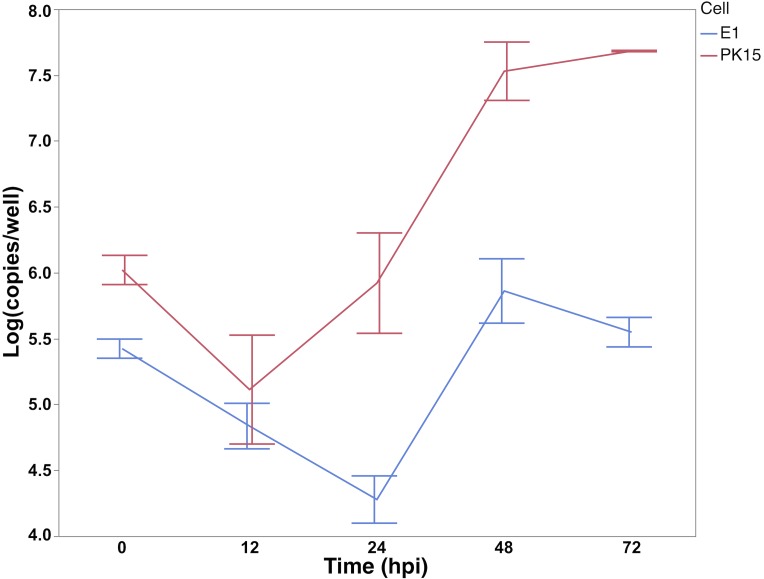
PCV2 copy number in E1 and wildtype PK15 cells following inoculation with the UNL2014001 PCV2b strain. The number of viral copies from PK15 cells is expressed as log10 copies/well.

**Fig 12:**
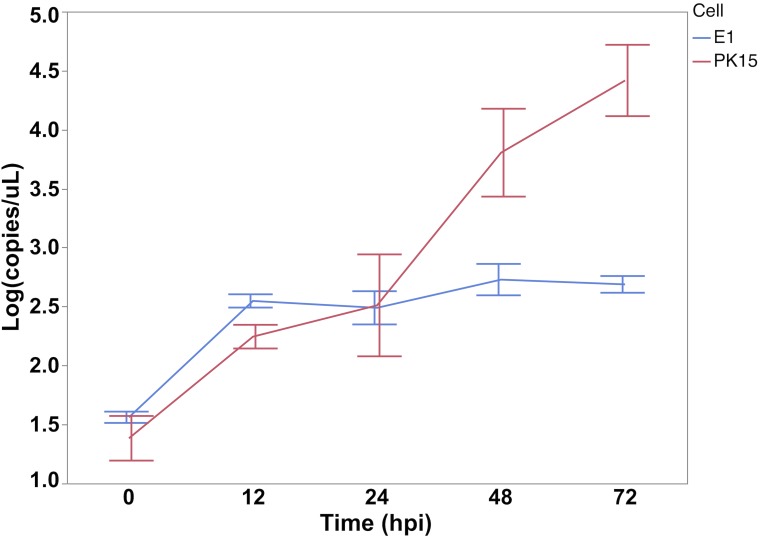
PCV2 copy number in the supernatant obtained from E1 and wildtype PK15 wells following inoculation with the UNL2014001 PCV2b strain. The number of viral copies is expressed as log10 copies/ul.

PK15 edited clones were generated by CRISPR-Cas9 Ribonucleoprotein (RNP) complex approach with a pair of guide RNAs (31_AC/40_AC) targeting the second exon of *SYNGR2* to cause a partial deletion of this exon and removal of the region containing the *SYNGR2 p*.*Arg63Cys* polymorphism (Fig 10). Sequencing of the mRNA from selected PK15 edited clones revealed a single clone homozygous for the same 106 bp deletion (*E1*). This deletion is predicted to cause a shift in the reading frame and an altered protein (195 residues) beginning at amino acid residue 42 compared to the wildtype SYNGR2 sequence (224 residues). The deleted fragment included the conserved motif located in the first loop while the shift in the reading frame affected the C-terminus of *SYNGR2*. A significant reduction in viral titer starting at 24 hpi in cells (Fig 11) and 48 hpi in supernatant (Fig 12) was observed in the E1 edited clone compared to wildtype PK15 (P < 0.05).

The induced changes resulted in a potential nonsense-mediated mRNA decay, since a nominal reduction in expression of *SYNGR2* was observed in E1 cells compared to wildtype PK15 cells with a significant difference observed at 24 hpi (P <0.05, S9 Fig).

## Discussion

Substantial variation in efficiency of viral replication and specific immune response was reported in our previous studies of experimental infections with PCV2 [7, 8]. Host genotype explained a substantial proportion of the phenotypic variation for viremia, viral load and immune response, with two major QTL identified on SSC7 and SSC12. Despite the presence of these two major loci, GWAS across time points following infection underlined the quantitative nature of the phenotypic variation of the targeted traits. The genetic complexity of PCV2 susceptibility has been augmented by the presence of a QTL in the vicinity of the SLAII complex of genes (SSC7). Despite the known role of this region in antigen recognition and immune response, high LD and genetic diversity have limited discovery of functional variants. Dissection of the SSC12 QTL based on gene annotation, genomic and RNA-sequencing uncovered a non-conservative substitution in a key domain of the *SYNGR2* gene associated with PCV2 viremia and immune response. SYNGR2 is a non-neural member of the synaptogyrin family, a group of genes primarily expressed in the membrane of synaptic vesicles of neuronal cells with roles in vesicle biogenesis, exocytosis and recycling via endocytosis [15, 18]. There is limited information about the functional role of this member of the gene family. Recently, *SYNGR2* was implicated as an active player in promoting viral RNA replication and immune evasion of severe fever with thrombocytopenia syndrome virus (SFTSV), a novel tick-borne bunyavirus in humans [17]. SYNGR2 interacted with non-structural viral proteins to promote the formation of lipid–based inclusion bodies, which become virus factories within the cytoplasm of infected cells. *SYNGR2* mRNA had been upregulated more than 200-fold at 36 hpi with SFTSV. *In vitro* silencing of *SYNGR2* resulted in a decrease in viral replication and a reduction in the number and size of the inclusion bodies, further substantiating the role of *SYNGR2* in facilitating SFTSV infection [17].

Similarly, our study showed that silencing the expression of *SYNGR2* in PK15 cells was associated with a significant reduction in PCV2 titer, indicating a role of *SYNGR2* in promoting viral replication. *SYNGR2 p*.*Arg63Cys*, the only missense polymorphism identified in *SYNGR2*, is characterized by a predicted change in charge and hydrophobicity of the first loop that connects two essential transmembrane domains, and is located in a region conserved across mammals. In rats, the first intraluminal loop and the C-terminus of SYNGR2 were found to be crucial for successful incorporation of the protein into vesicular membranes and vesicle formation [15]. Replacement of residues 67–73 in the first loop led to protein degradation, with residues 70–73 having the largest impact [15]. In pigs, this segment of four residues is analogous to amino acids 60–63 and identical with the rat sequence (Val-Phe-Asn-Arg) corresponding to *SYNGR2 p*.*63Arg* allele (Fig 4). Since the *SYNGR2 p*.*Arg63Cys* substitution is located within this crucial region, we hypothesize that *SYNGR2 p*.*63Cys* allele could influence incorporation of SYNGR2 into vesicular membranes, impact vesicle formation, and efficient trafficking of PCV2 to nucleus for replication. Using CRISPR-Cas9 that targeted the second exon which encodes this important motif, we generated a PK15 *SYNGR2* edited clone. A reduction in viral titer observed in the edited clone, underlined the critical role of this gene in PCV2 susceptibility.

Predominance of the *SYNGR2 p*.*63Cys* allele in Pietrain and Duroc compared to other domestic and wild pig breeds could be a result of the subclinical effects that early PCV2 strains had on growth prior to the surge in PMWS in the early 1990’s and the emphasis on growth in selection of these two breeds. In the Danish pig breeding program, five times more emphasis is placed on ADG (30–100 kg) in Duroc than in Large White or Landrace (Bolhom, 2010, personal communication; http://docplayer.net/20998319-Danish-pig-production.html). This selection pressure and the presence of mild PCV2 strains could have resulted in a rapid increase in the frequency of the *SYNGR2 p*.*63Cys* allele in both Pietrain and Duroc.

In our research we did not observe an increase in *SYNGR2* mRNA levels following *in vitro* or *in vivo* infection with PCV2. This may reflect important distinctions between SFTSV and PCV2. For instance, SFTSV is an RNA virus with the capacity to replicate within intracytoplasmic inclusion bodies, or viral factories. As Sun et al. (2016) demonstrated, SYNGR2 is a component of these vesicles and necessary for their formation [17]. As SFTSV replicates, more viral factories will be required for viral proliferation, resulting in increased levels of SYNGR2. PCV2, on the other hand, is a DNA virus and can only replicate in the nucleus of host cells. Therefore, the potential role of SYNGR2 in PCV2 infection likely takes place prior to viral replication and may not require such an increase in SYNGR2 expression, but rather specific SYNGR2-PCV2 interactions. Since the position of this substitution is clearly located in a loop and not part of the transmembrane regions indicated by Janz and Sudhof (1998) and predicted by PSIPRED [19] (S10 and S11 Figs), an interaction between SYNGR2 and a ligand is favored compared to the potential impact of *SYNGR2 p*.*Arg63Cys* on overall protein folding or conformation of the first loop. A shift in the position of the second transmembrane helix as a result of the substitution in the loop region was predicted by HMMTOP software [20], but not supported by others (e.g., TMHMM, DisEMBL, PSIPRED).

In this study, decreased viral titer by 1) exogenous reduction of *SYNGR2* expression by siRNA along with 2) partial deletion of a key domain by gene editing, provided evidence of the involvement of *SYNGR2* in PCV2 infection. Since the *SYNGR2 p*.*Arg63Cys* polymorphism is the only missense mutation within the entire gene and located in this key domain, it is a plausible QTN (Quantitative Trait Nucleotide) candidate for PCV2 susceptibility. However, future studies of the mechanistic role of *SYNGR2* and specifically of *SYNGR2 p*.*Arg63Cys* substitution will be required to provide additional experimental evidence of their role in PCV2 replication and pathogenesis.

## Materials and methods

### Ethics statement

The experimental design and procedures used during this research project were approved by the Institutional Animal Care and Use Committee of the University of Nebraska -Lincoln.

### Experimental design: Animals, diets, and housing

Experimental PCV2b challenge was conducted in nine batches that varied in size from 81 to 141 pigs, with a total of 974 pigs. The genetic makeup of this resource population consisted of crossbred pigs produced by 14 genetic lines generated by seven genetic programs. The dams of the experimental pigs had been vaccinated for PCV2 at 3 weeks of age with a single dose of Ingelvac CircoFLEX vaccine (Boehringer Ingelheim). The suppliers of the pigs also had vaccination programs for Porcine parvovirus, *Erysipelothrix rhusiopathiae*, *Clostridium perfringens*, Leptospirosis and Colibacillosis and tested negative for Porcine Reproductive and Respiratory Syndrome Virus (PRRSV). Prior to experimental infection, the pigs tested negative for presence of PCV2 in peripheral blood by real time quantitative PCR (qPCR) and had a sample/positive ratio (S/P) lower than 0.4 for IgM and 0.3 for passive IgG, the PCV2-specific antibodies [7]. Following infection, experimental pigs were examined daily for clinical signs of disease; weights and blood samples were collected at 0, 7, 14, 21 and 28 days post infection (dpi). Details of the experimental procedures, phenotypic and sample collection are described in Engle et al. (2014) and McKnite et al. (2014).

A validation dataset consisting of a group of 71 pigs representing all three *SYNGR2 p*.*Arg63Cys* genotypes infected with the same PCV2b strain at 5 weeks of age was generated using the same experimental conditions. A group of 40 pigs (*SYNGR2 p*.*63Arg/63Cys* and *63Arg/63Arg*) vaccinated for PCV2 at 3 weeks of age were used as controls. The vaccinated pigs were housed in the same room with the experimentally infected pigs, but in different pens.

### PCV2b isolate and experimental infection

The PCV2b strain (UNL2014001) used for the experimental infection was obtained from a pig with symptoms characteristic to Post-weaning Multisystemic Wasting Syndrome (PMWS), which is the most common PCVAD syndrome. The strain was sequenced (accession KP016747.1) using dye terminators and the sequence was compared to PCV2 strains available in GenBank [8]. The strain was cultured in swine testicular cell lines as described [7]. At an average of 36 d all the pigs were inoculated with the UNL2014001 PCV2b strain with a titer of 10^4.0^ 50% tissue culture infection dose (TCID50) intranasally and intramuscularly.

### Serologic profile: Quantification of viral DNA and PCV2-specific antibodies

PCV2 specific antibodies, IgM and IgG, were profiled weekly from serum using ELISA (Ingenasa) as described in McKnite et al. (2014). Estimates of the number of PCV2b copies, or viremia, was performed using viral genomic DNA isolated by QIAamp DNA Minikit (Qiagen) and quantified by qPCR using TaqMan Master Mix and ABI 7900 Real Time PCR System (Thermo Scientific). The viral load for each pig during the entire challenge was represented as area under the curve (AUC) based on an algorithm that takes into account viral levels observed at each time point following infection (0, 7, 14, 21, and 28 dpi) fitting a smooth curve over the 28 days and summing the areas in 0.01 time increments [21].

### Host genetic profile: DNA isolation, sequencing, polymorphism discovery and genotyping

The DNA was isolated from ear and tail tissue clips using DNeasy or Puregene blood and tissue kits (Qiagen). The experimental animals were genotyped using either the first or second generation of the Porcine SNP60 BeadArray (Illumina) that contain 62,183 and 61,565 SNPs, respectively. Only the common SNPs present in both BeadArray versions (91.6%, 61,177) were mapped on *Sscrofa* 11.1 porcine reference genome assembly and used in GWAS via GenSel software package [22]. DNA samples and SNP assays with a genotyping call rate below 80% were excluded from the analyses. A GenCall quality score of 0.40 was used as a minimum threshold for genotype quality [23].

Targeted DNA sequencing of candidate genes in the SSC12 QTL region including *SOCS3*, *BIRC5 and SYNGR2* and their 2–4 kb region upstream of the transcription start sites (TSS) was performed using dye terminators and ABI PRISM 3100 Genetic Analyzer (Thermo Scientific) on high and low viremic samples. Discovery and validation of the polymorphisms detected by RNA-seq was based on alignment of DNA sequences using Sequencher software (Gene Codes). Potential impact of the polymorphisms located in the proximal promoter on important regulatory motifs was evaluated using FIMO (version 4.11.3) [24] and the JASPAR transcription profile database (version 2016).

Genotyping of polymorphisms located in the transcribed regions and proximal promoters of *SOCS3*, *BIRC5*, *SYNGR2*, *THA1*, *TMC6* and *TMC8* was performed by multiplex assays using Sequenom MassARRAY platform and Sequenom iPLEX chemistry based on the manufacturer protocols (Sequenom, San Diego, CA).

### Genome-wide associations

The proportion of phenotypic variance explained by host genetics for PCV2-viremia, PCV2-specific antibodies (IgM and IgG) and average daily gain (ADGi) during experimental infection was estimated based on Porcine SNP60 BeadArray genotypes using a BayesB model [25] and GenSel software [22]. The statistical model included litter, pen and batch as class variables and passive IgG and age at infection as covariates. Bayesian analyses were based on *π* equal to 0.99 that assumed a prior probability of 1% of the SNPs having a non-zero effect. The Markov chain included 40,000 samples with the first 1,000 being removed as burn-in. Markov chain was set to use every 40^th^ sample to estimate posterior distribution for the genetic variance explained by each 1 Mb window of the reference genome. This distribution was used to estimate the probabilities of each 1Mb window having a variance greater than 0 or greater than the average variance explained by each 1Mb window as described in McKnite et al. (2014).

Bayes Interval Mapping (BayesIM) was implemented to derive haplotype effects across the genome on PCVAD-related traits as described in Kachman (2015) and Wilson-Wells and Kachman (2016) [11]. Briefly, a hidden Markov model was used to generate 8 haplotype states based on SNP genotypes [26]. Phenotypic variation of the targeted traits was analyzed with a hierarchical Bayesian model. QTL were placed every 50 kb across the genome while average haplotype size was set to 500 kb. Genetic variances, haplotype effects, and model frequencies were estimated at each locus. There were 42,000 MCMC samples collected with the first 2,000 used for burn-in. The model included batch, litter and pen as random effects and IgG and age at infection used as covariates. If a locus had an effect, haplotype effects for each cluster were modeled as independent normal random variables.

Associations between the single marker genotypes and phenotypic variation were tested using a linear mixed model fitted by JMP 10.0 (SAS Inst. Inc.) that included marker genotype and batch as fixed effects, litter and pen as random effects while age at infection and IgG were used as covariates. Additive and dominance effects were estimated for each of the targeted DNA polymorphisms. A similar model was used to estimate the interaction between SNPs. The potential effect on viral load of the haplotypes in the defined LD block from *ALGA0110477* to *SYNGR2* that includes 16 DNA polymorphisms, was estimated as haplotype substitution effects. Contrasts between haplotypes were estimated using a linear mixed model as described above including one variable for each haplotype with values 0, 1, and 2 corresponding to the animal having 0, 1, or 2 copies of the haplotype in question. The haplotype substitution effects were presented as deviations from the mean of the haplotypes.

### Novel assembly and annotation of the proximal end of SSC12

Inverse PCR (iPCR), using four (AciI, AluI, HaeIII, HpaII, RsaII) and six cutter (EcoRI, HaeII, HincII, HindIII, KpnI, MfeI, MspA1l) restriction enzymes (New England Biolabs), T4 DNA ligase (New England Biolabs) and nested PCR using AmpliTaq Gold 360 DNA polymerase (Thermo Scientific), was employed to expand the genomic DNA sequence surrounding the short *ALGA0110477* sequence, a SNP previously unmapped on the *Sscrofa* 10.2 reference genome. A genomic scaffold (19 Mb) of the proximal end of SSC12 was constructed based on Pacific Biosciences sequencing reads [11]. The position of the extended *ALGA0110477* sequence and all SSC12 mapped and unmapped SNPs were determined on the genomic scaffold using BLAT. Annotation of the QTL region on the SSC12 scaffold was based on RNA-seq alignments and BLAST but also using *ab initio* approaches such as GenScan [13, 27] in combination with pBLAST.

### RNA-seq and gene expression profiling

In order to profile transcriptome changes and sequence variation related to PCV2 infection, peripheral blood samples collected from the validation group of pigs that exhibited high (N_*TT*_ = 6) and low (N_*CC*_ = 5) viremic genotypes for *ALGA0110477* at 0, 7 and 14 dpi were subjected to RNA sequencing. RNA was extracted from peripheral blood collected in Tempus tubes using the Tempus Spin RNA Isolation Reagent Kit (Thermo Scientific). RNA samples were sequenced using Ion Proton technology as described in the manufacturer protocol (Thermo Fisher Scientific Inc.). The adaptor-free sequencing reads were trimmed and filtered using Trim galore (version 0.4) [28] with low-quality bases in the 5’ end being removed and nucleotides with quality call less than 22 being trimmed from the 3’ end. The filtered reads were initially aligned to the SSC12 scaffold (19 Mb) using the two-step alignment approach used for Ion Proton transcriptome data that includes both Tophat and local-Bowtie [29]. The reads were later also aligned to the new pig assembly *Sscrofa* 11.1. The number of reads mapped to each gene in the annotated QTL region was obtained using HTSeq (version 0.6.1p1) [30].

Expression of the candidate genes *SOCS3*, *BIRC5* and *SYNGR2* across time points following PCV2 infection was quantified using TaqMan Master Mix and CFX384 Real Time PCR (BioRad). The qPCR assays were designed using IDT Realtime PCR Tool software (www.idt.com) and sequences generated based on RNA-seq alignments. RNA was extracted from peripheral blood samples collected in Tempus tubes from a subset of pigs representing all genotypes from the validation data set that displayed extreme viral load (high vs low) (n = 40) from 0 to 21 dpi using the Tempus Spin RNA Isolation Reagent Kit (Thermo Scientific). Complementary DNA (cDNA) was obtained using a mix of random hexamers and poly dT primers using First strand cDNA Synthesis Kit (GE Healthcare Bio-Sciences). Expression of ribosomal protein L32 (*Rpl32*) gene was used for normalization. Mean normalized expression (MNE) values were calculated based on cycle crossing thresholds (CT) obtained for the technical triplicates taking qPCR efficiencies into account [31]. MNE values for *ALGA0110477*, *SYNGR2 p*.*Arg63Cys* or *BIRC5 g*.*-343delA* genotypes and time point following infection were log10 transformed and compared by t-test.

### *In vitro* PCV2 infection of PK15 cells

The porcine kidney cell line (PK15) was grown in DMEM high glucose media supplemented with 10% FBS and 1% Penicillin-Streptomycin (5,000 U/mL). Cells were cultured in 12-well plates (4 cm^2^) with 5.0x10^5^ cells per well and infected with UNL2014001 PCV2b strain (TCID_50_ = 10^4^) when 80–100% confluent at MOI = 0.00025. One hour following infection, cells were washed and fresh media was added (DMEM high glucose and 2% FBS). The cells were incubated at 37 °C with 5% CO_2_ for up to 5 days. Control cells were maintained the same way and mock-inoculated with plain DMEM high glucose media. Supernatants and cells were collected at specific time points and frozen at −80 °C. Viral DNA was extracted from supernatants using QIAamp DNA Mini kit (Qiagen). RNA, viral and host DNA was extracted from PK15 cells using AllPrep DNA/RNA Mini kit (Qiagen). TaqMan Master Mix and CFX384 Real Time PCR Detection System were used for quantification of PCV2 and expression profiling of *BIRC5* and *SYNGR2* from PK15 cells. Dideoxy sequencing of the cDNA and genomic DNA was used to profile the sequences and to genotype *BIRC5* and *SYNGR2* variants in PK15 cells.

### *In vitro* silencing of *SYNGR2* in PK15 cells

PK15 cells were transfected 24 hours after plating in 12-well plates (4 cm^2^) with 2.5x10^5^ cells per well when ~80% confluent with two siRNA oligos (siRNA-01: sense 5’-CUACAAGGCCGGAGUGGAUUU-3’, and antisense 5’-AUCCACUCCGGCCUUGUAGUU-3’; siRNA-03: sense 5’-CCACAAGUCCGGAGAGCAGUU 3’, and antisense, 5’-CUGCUCUCCGGACUUGUGGUU-3’, Dharmacon Research) targeting *SYNGR2* mRNA and the AllStars Negative Control siRNA (scramble, Qiagen) at 10nM and 20nM concentrations. Transfection was performed using Lipofectamine RNAiMAX transfection reagent (Invitrogen) following the manufacturer’s protocol. Cell samples were collected 24, 48, 72, and 96 hours post transfection in PBS and centrifuged at 16,000xg for 1 minute to pellet the cells. RNA was extracted using RNAeasy Mini kit (Qiagen). Real Time PCR was used to profile SYNGR2 expression. siRNA oligo 01 and the AllStars Negative Control siRNA (20nM) were used for subsequent transfections prior to infection. The siRNA transfected cells were inoculated 24 hr after transfection following the same infection protocol described above. Statistical differences in viral titer between cell lines across time points were tested using a linear model fitted by JMP 10.0 (SAS Inst. Inc.) that included batch and cell line as fixed effects. Pairwise comparisons between least-squares means of the viral titers were based on the Tukey test.

### *In vitro* editing of *SYNGR2* in PK15 cells

Six potential guide RNAs targeting the second exon of *SYNGR2* were designed and ordered (IDT), three located upstream (5’) and three located downstream (3’) of the *SYNGR2 p*.*Arg63Cys* polymorphism. Each guide RNA was hybridized with fluorescently labeled Alt-R CRISPR-Cas9 tracrRNA ATTO 550 (IDT) and Alt-R S.p. Hifi Cas9 Nuclease V3 (IDT) following the manufacturer’s protocol to form Ribonucleoprotein (RNP) complexes. These RNP complexes were reverse transfected into PK15 cells using Lipofectamine RNAiMAX transfection reagent (Invitrogren) at a final concentration of 10nM. After 48 hours post transfection, genomic DNA was extracted using QIAamp Blood DNA Mini Kit (Qiagen) and amplified via PCR using LongAmp Taq DNA polymerase (NEB) with primers located in the introns flanking the second exon of *SYNGR2* (5’-AGAAGGGAGAGACAGCACCA-3’, 5’- CACCAGCACATCTTCCACCT-3’). The amplicons were subjected to T7 endonuclease I (NEB) digestion following the manufacturer’s protocol and visualized by agarose gel electrophoresis to assess cutting efficiency of each individual guide RNA. The ability of guide RNA pairs (upstream/downstream) to generate partial deletions of the second exon was assessed following the same RNP transfection protocol with a final RNP concentration of 20nM (10nM/guide RNA). After 48 hours post transfection, genomic DNA was extracted, amplified, and visualized via agarose gel electrophoresis.

A single guide RNA pair (sg31_AC/sg40_AC) was selected to generate PK15 edited clones. After 24 hours post transfection, the cells were collected and sorted using Fluorescence Activated Cell Sorting (FACS) into 96 well plates to generate single cell clones. Genomic DNA from each single cell clone was extracted using QuickExtract DNA extraction solution (Lucigen) following the manufacturer’s protocol and genotyped by PCR amplification and gel electrophoresis. RNA was extracted from selected clones using All Prep DNA/RNA Mini kit (Qiagen) and PCR was performed with primers located in the 5’ and 3’ UTRs of the *SYNGR2* mRNA sequence (5’-ACGGCGACAATGGAGAGCGG-3’, 5’-GGGAAACAAGAGGGGCCAGCA-3’) to amplify full-length transcripts, which were sequenced using Dideoxy sequencing. A single clone (*E1*) homozygous for a 106bp deletion was plated and infected with PCV2b inoculate as previously described. Wildtype PK15 cells were concurrently infected and served as a control.

To determine if induced changes in the mRNA sequence of *SYNGR2* led to nonsense-mediated mRNA decay, expression of *SYNGR2* was profiled by qPCR at 0, 24 and 48 hrs in edited and wildtype PK15 cells.

## Supporting information

S1 Fig Genome-wide association between 56,557 SNPs and PCV2b viral load using BayesB. Each dot represents the proportion of genetic variance explained by an individual SNP. The x-axis represents the position of each SNP in the swine genome using *Sscrofa* 11.1 assembly. The y-axis represents the contribution of each SNP to the genetic variance. Alternate colors represent autosomes, from SSCs 1 to 18, chromosome X and Y followed by a set of SNPs without a genomic location.
(TIF)

S2 Fig Genome-wide association between Porcine SNP60 BeadArray genotypes and PCV2b viral load using BayesIM. Alternate colors represent autosomes, from SSC1 to 18. Unmapped SNPs in the previous *Sscrofa* 10.2 assembly including *ALGA0110477* were excluded from the analysis. Each dot represents the model frequency associated with 50kb QTL. The X-axis represents the position of the 50 kb loci across the swine genome using *Sscrofa* 10.2 assembly. The Y-axis represents the model frequency of the association between a QTL and PCV2b viral load.
(TIF)

S3 Fig Haplotype effects for PCV2b viral load across the proximal end of SSC12 estimated using BayesIM model. (TIF)

S4 Fig Genome-wide association between 51,592 SNPs and PCV2b viremia using BayesIM. Each dot represents the model frequency associated with each 50kb QTL. The X-axis represents the position of the 50 kb loci across the swine genome using *Sscrofa* 11.1 assembly. The Y-axis represents the model frequency of the association between a QTL and PCV2 viremia. Alternate colors represent autosomes, from SSC1 to 18.
(TIF)

S5 Fig Genome-wide association between 51,592 SNPs and PCV2-specific IgM using BayesIM. Each dot represents the model frequency associated with each 50kb QTL. The X-axis represents the position of the 50 kb loci across the swine genome using *Sscrofa* 11.1 assembly. The Y-axis represents the model frequency of the association between a QTL and IgM following PCV2 infection. Alternate colors represent autosomes, from SSC1 to 18.
(TIF)

S6 Fig Genome-wide association between 51,592 SNPs and PCV2-specific IgG using BayesIM. Each dot represents the model frequency associated with each 50kb QTL. The X-axis represents the position of the 50 kb loci across the swine genome using *Sscrofa* 11.1 assembly. The Y-axis represents the model frequency of the association between a QTL and IgG following PCV2 infection. Alternate colors represent autosomes, from SSC1 to 18.
(TIF)

S7 Fig Least square means and standard errors of the *SYNGR2 p*.*Arg63Cys* genotypes (*63Cys/63Cys* -green, *63Arg/63Cys*-red, *63Arg/63Arg*-blue) across weekly IgM PCV2-specific antibody following PCV2b challenge (n = 268). (TIF)

S8 Fig Least square means and standard errors of the *SYNGR2 p*.*Arg63Cys* genotypes (*63Cys/63Cys* -green, *63Arg/63Cys*-red, *63Arg/63Arg*-blue) across weekly IgG PCV2-specific antibody following PCV2 challenge (n = 268). (TIF)

S9 Fig Expression of *SYNGR2* in E1 and wildtype PK15 uninfected control cells. (TIFF)

S10 Fig Secondary structure of *SYNGR2 p*.*63Cys* allele predicted by PSIPRED v3.3 (http://bioinf.cs.ucl.ac.uk/psipred/). (TIFF)

S11 Fig Secondary structure of *SYNGR2 p*.*63Arg* allele predicted by PSIPRED v3.3 (http://bioinf.cs.ucl.ac.uk/psipred/). (TIFF)

S1 Table Genetic variance explained by 1Mb windows and 56,557 SNPs and PCV2b viral load using BayesB. (XLSX)

S2 Table Haplotype frequency and haplotype substitution effect for PCV2b viral load. (DOCX)
