# Supplementary figures and images for: Synaptogyrin-2 influences replication of Porcine circovirus 2
Source: PLoS Genet. 2018 Oct 31;14(10):e1007750. doi: 10.1371/journal.pgen.1007750 (PMC6245838; doi:10.1371/journal.pgen.1007750)

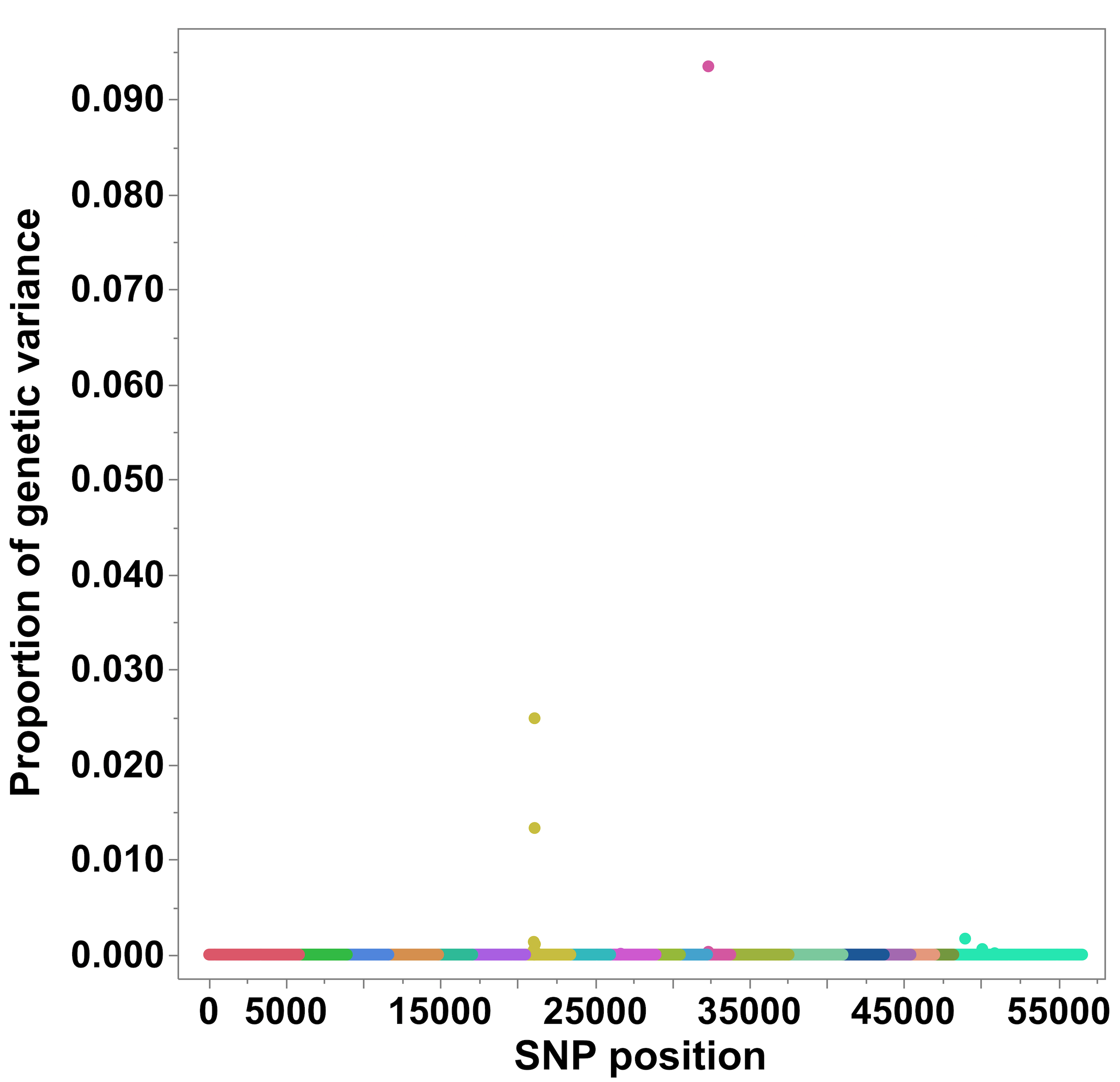

Supplement: S1 Fig — Each dot represents the proportion of genetic variance explained by an individual SNP. The x-axis represents the position of each SNP in the swine genome using Sscrofa 11.1 assembly. The y-axis represents the contribution of each SNP to the genetic variance. Alternate colors represent autosomes, from SSCs 1 to 18, chromosome X and Y followed by a set of SNPs without a genomic location. (TIF) [file pgen.1007750.s001.tif]

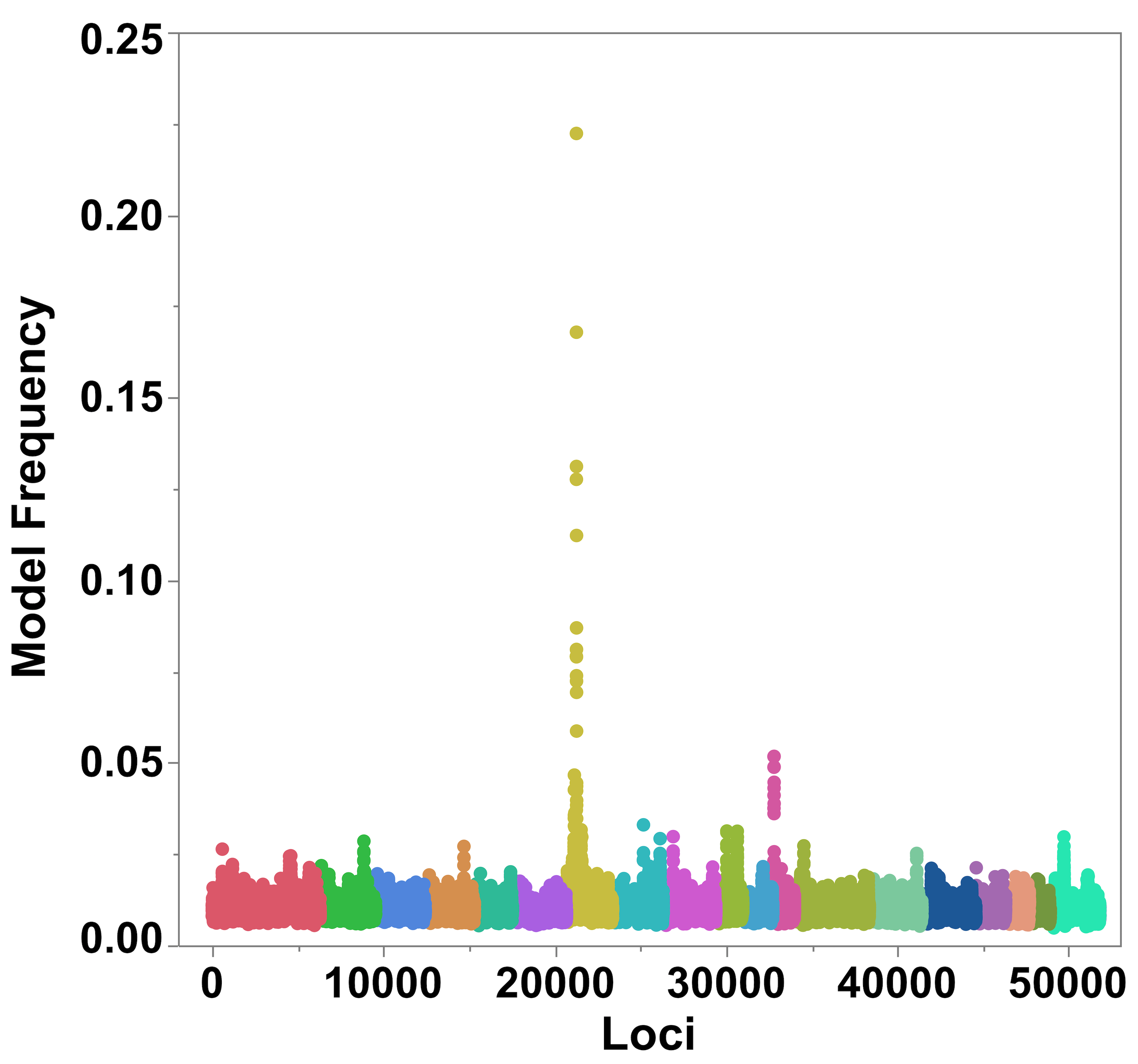

Supplement: S2 Fig — Alternate colors represent autosomes, from SSC1 to 18. Unmapped SNPs in the previous Sscrofa 10.2 assembly including ALGA0110477 were excluded from the analysis. Each dot represents the model frequency associated with 50kb QTL. The X-axis represents the position of the 50 kb loci across the swine genome using Sscrofa 10.2 assembly. The Y-axis represents the model frequency of the association between a QTL and PCV2b viral load. (TIF) [file pgen.1007750.s002.tif]

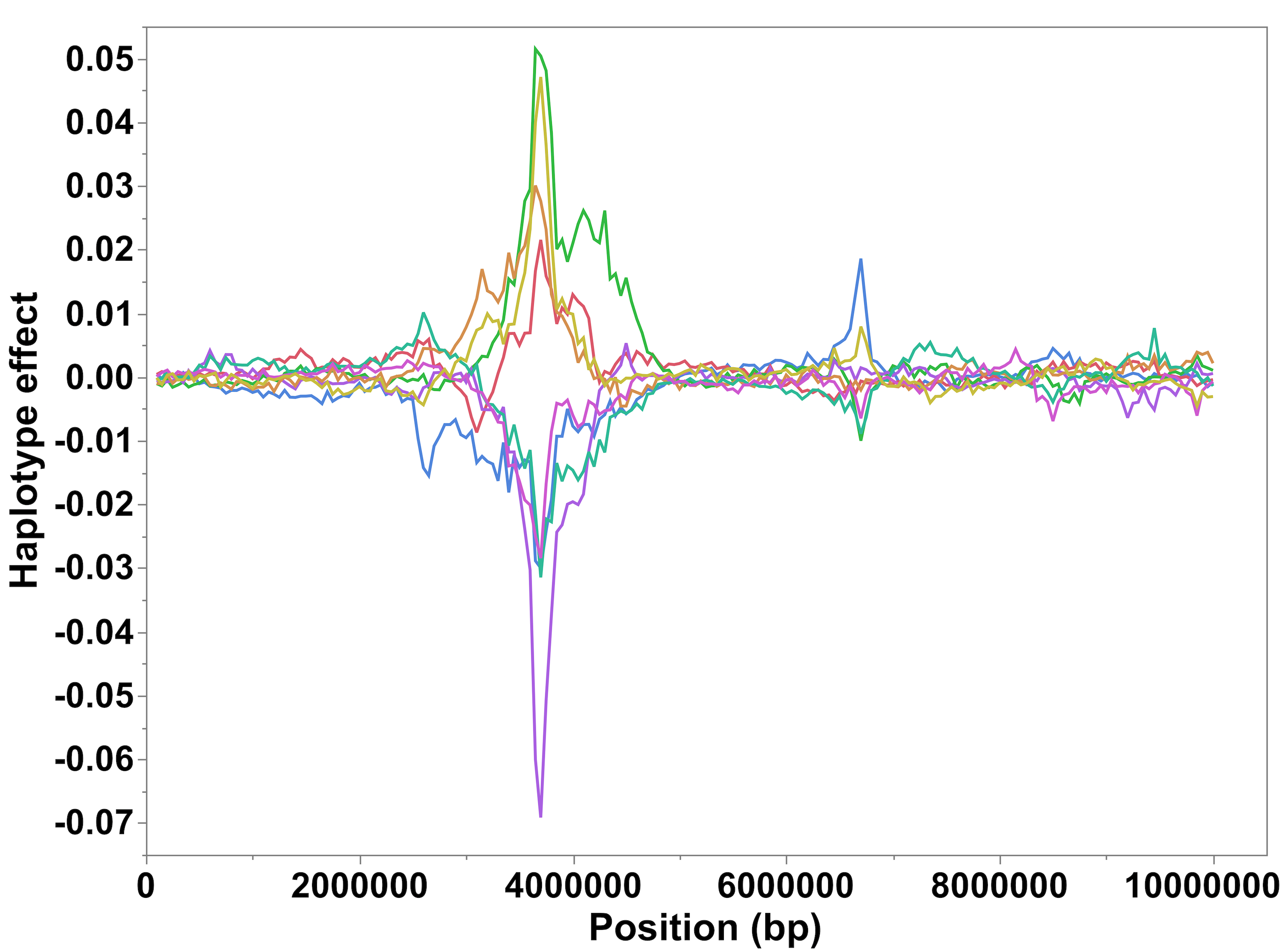

Supplement: S3 Fig — (TIF) [file pgen.1007750.s003.tif]

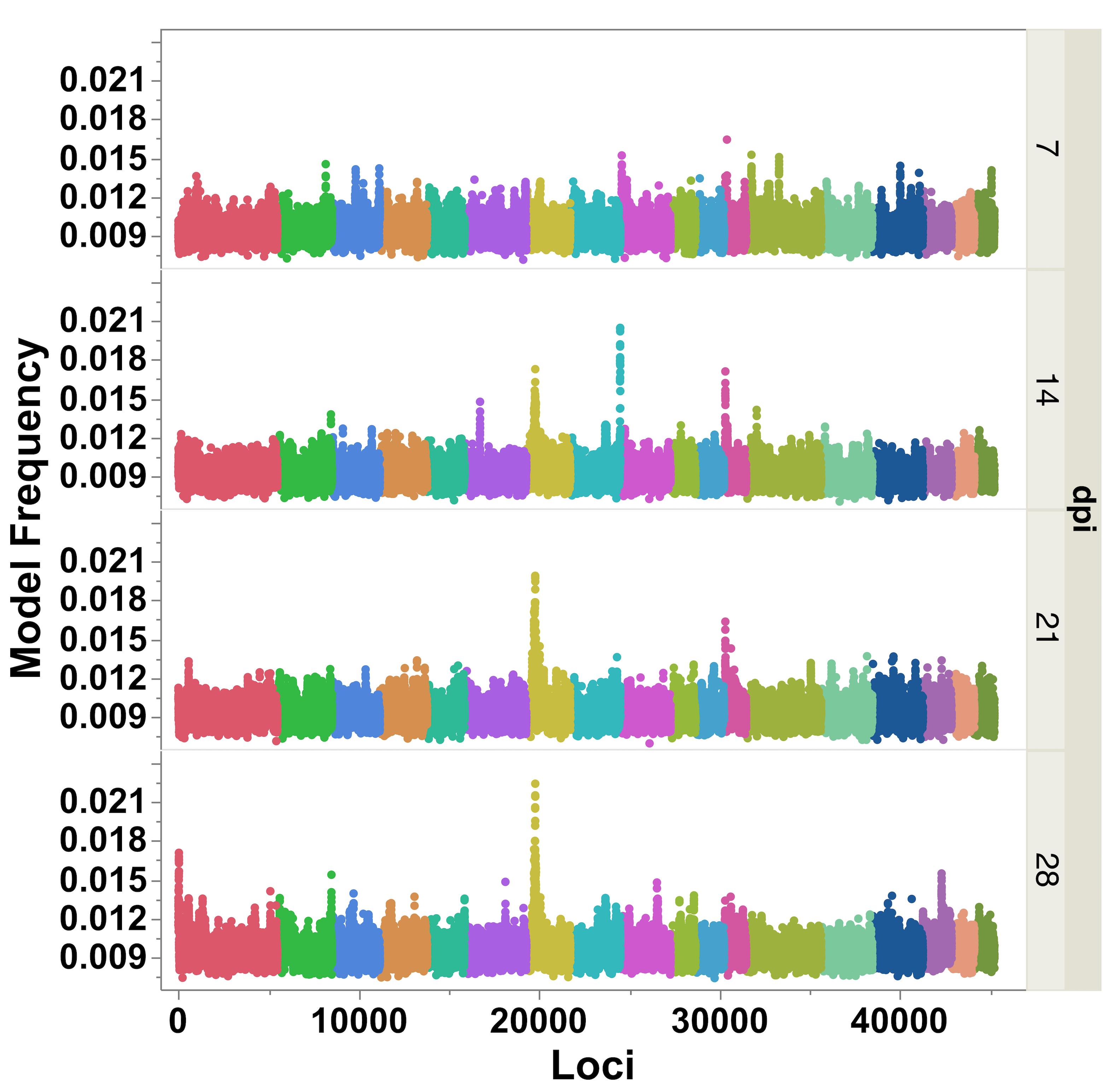

Supplement: S4 Fig — Each dot represents the model frequency associated with each 50kb QTL. The X-axis represents the position of the 50 kb loci across the swine genome using Sscrofa 11.1 assembly. The Y-axis represents the model frequency of the association between a QTL and PCV2 viremia. Alternate colors represent autosomes, from SSC1 to 18. (TIF) [file pgen.1007750.s004.tif]

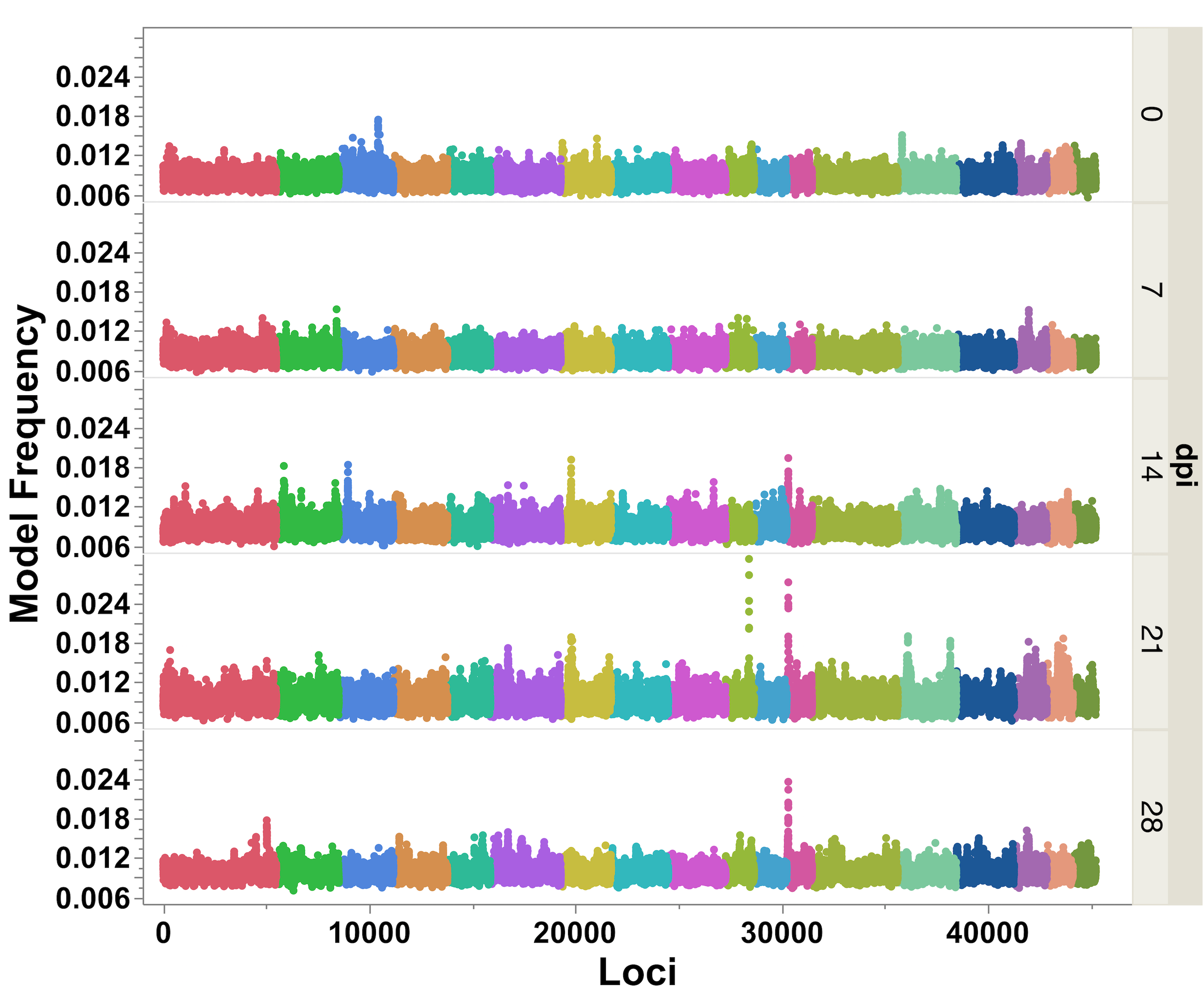

Supplement: S5 Fig — Each dot represents the model frequency associated with each 50kb QTL. The X-axis represents the position of the 50 kb loci across the swine genome using Sscrofa 11.1 assembly. The Y-axis represents the model frequency of the association between a QTL and IgM following PCV2 infection. Alternate colors represent autosomes, from SSC1 to 18. (TIF) [file pgen.1007750.s005.tif]

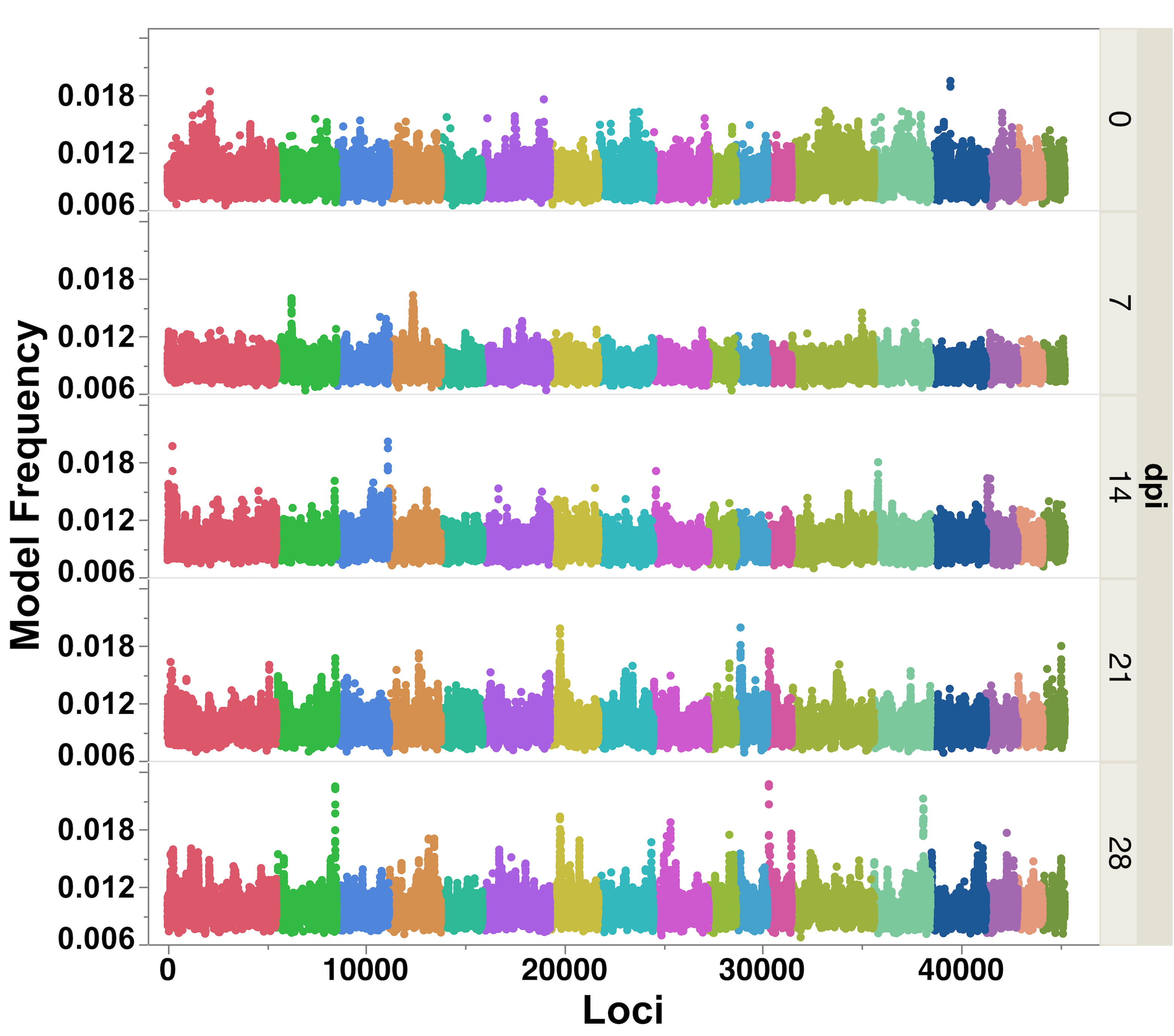

Supplement: S6 Fig — Each dot represents the model frequency associated with each 50kb QTL. The X-axis represents the position of the 50 kb loci across the swine genome using Sscrofa 11.1 assembly. The Y-axis represents the model frequency of the association between a QTL and IgG following PCV2 infection. Alternate colors represent autosomes, from SSC1 to 18. (TIF) [file pgen.1007750.s006.tif]

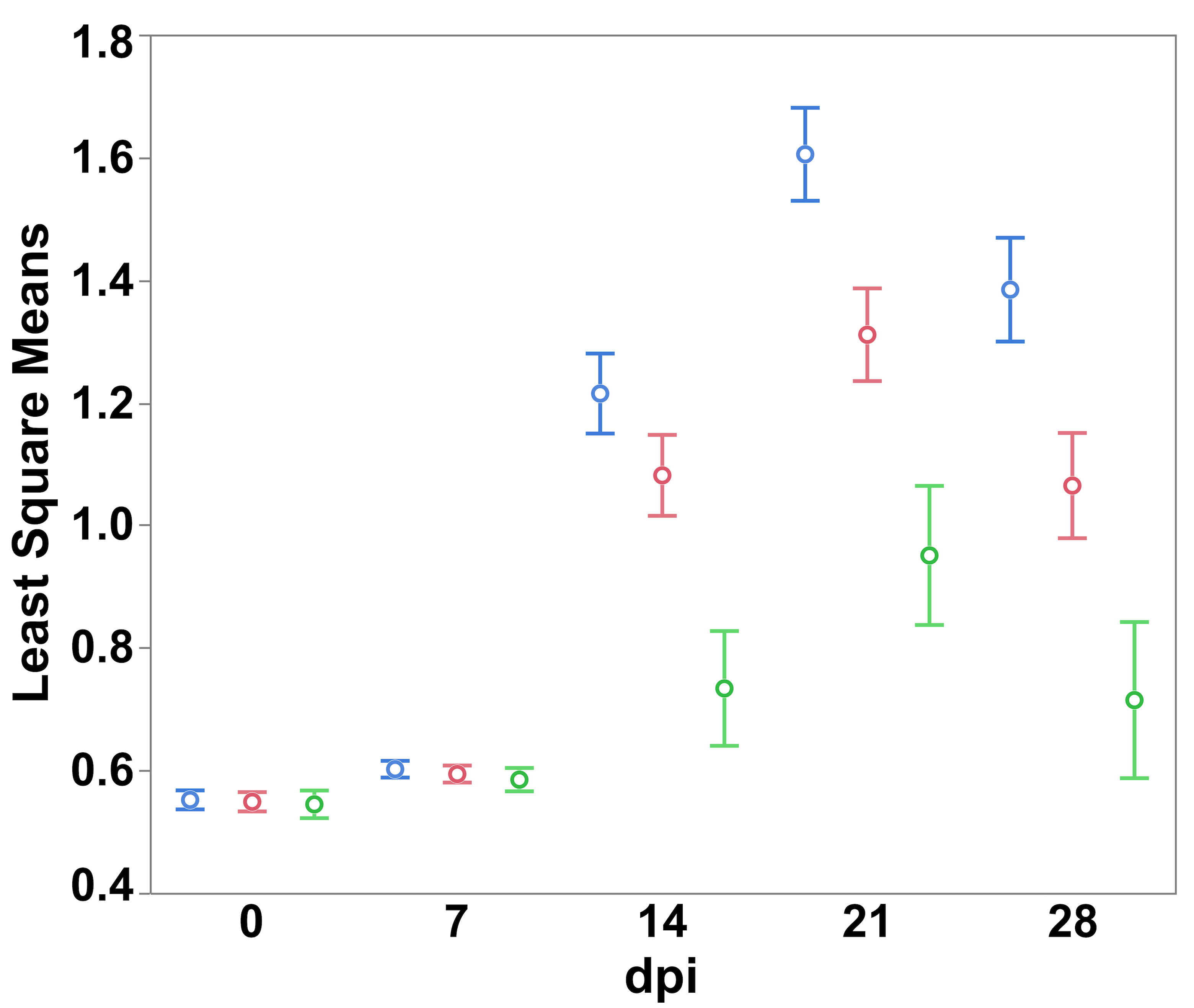

Supplement: S7 Fig — (TIF) [file pgen.1007750.s007.tif]

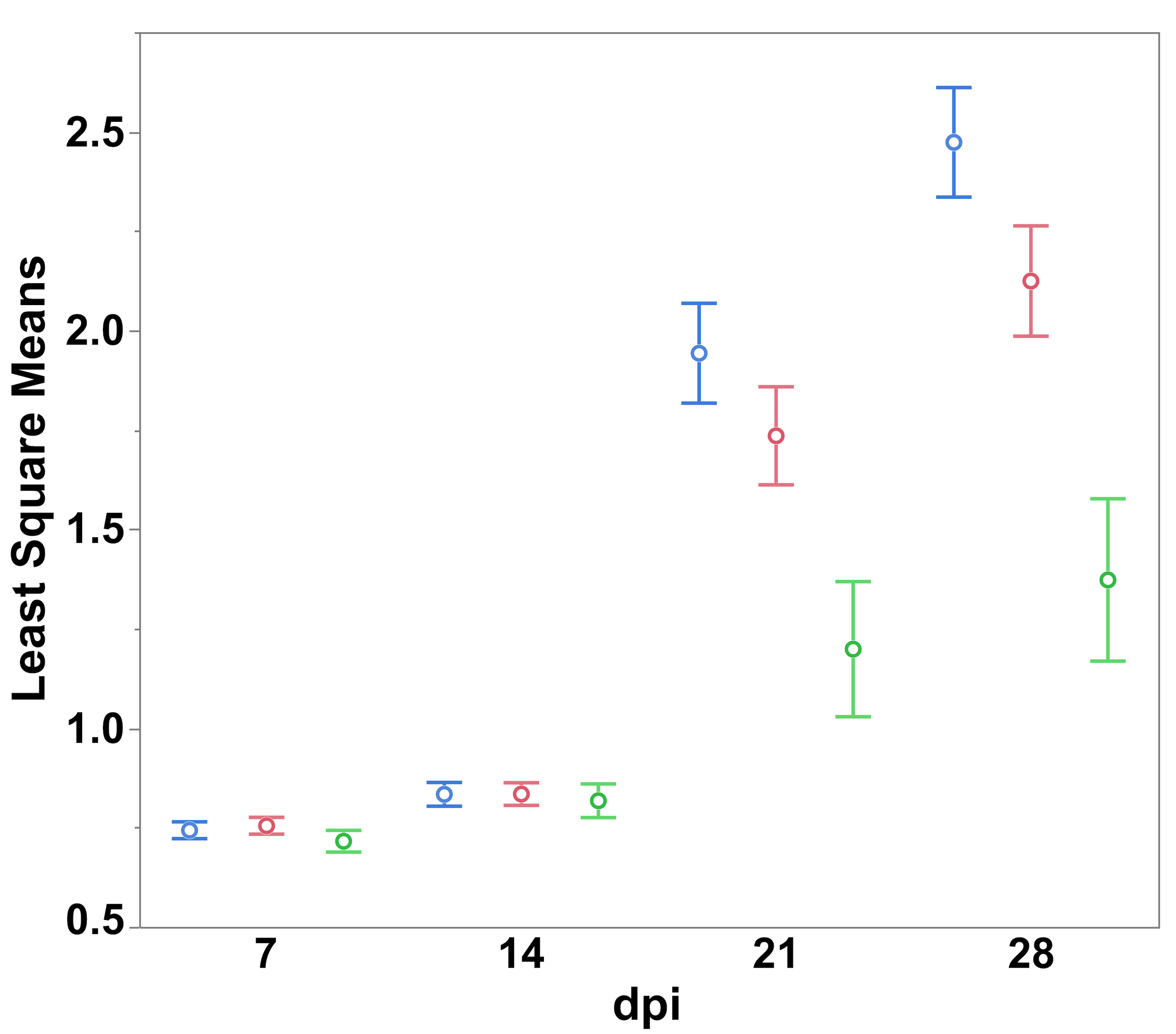

Supplement: S8 Fig — (TIF) [file pgen.1007750.s008.tif]

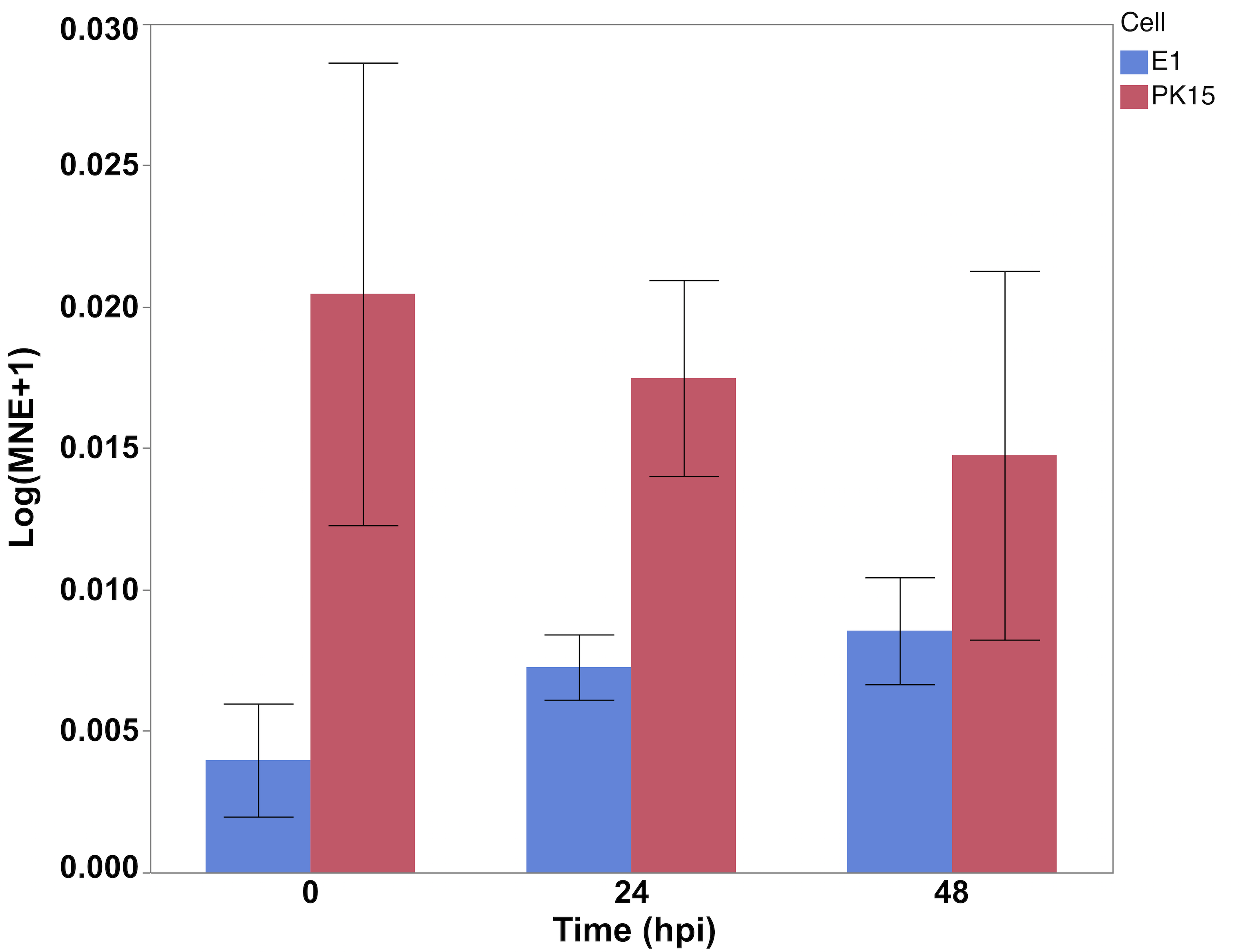

Supplement: S9 Fig — (TIFF) [file pgen.1007750.s009.tiff]

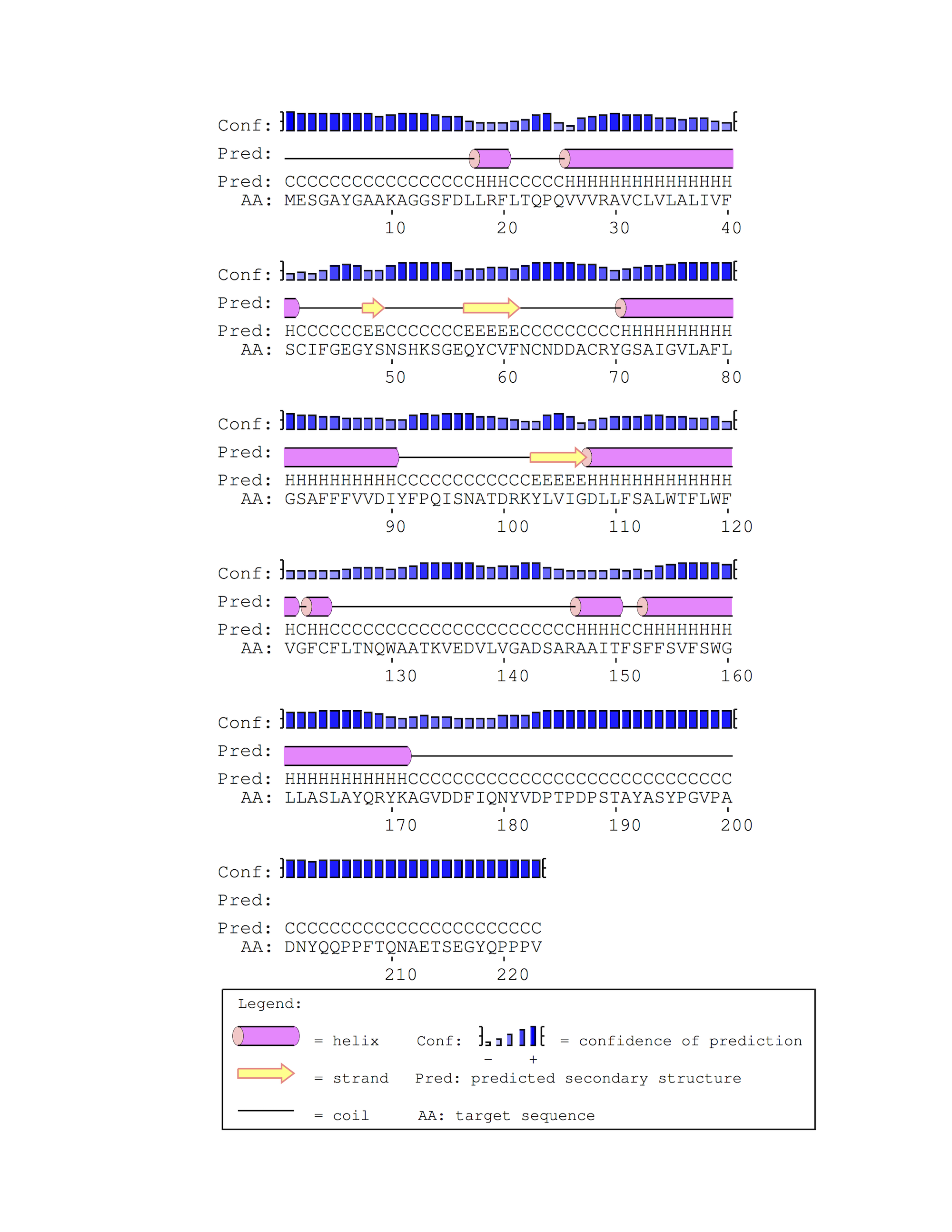

Supplement: S10 Fig — (TIFF) [file pgen.1007750.s010.tiff]

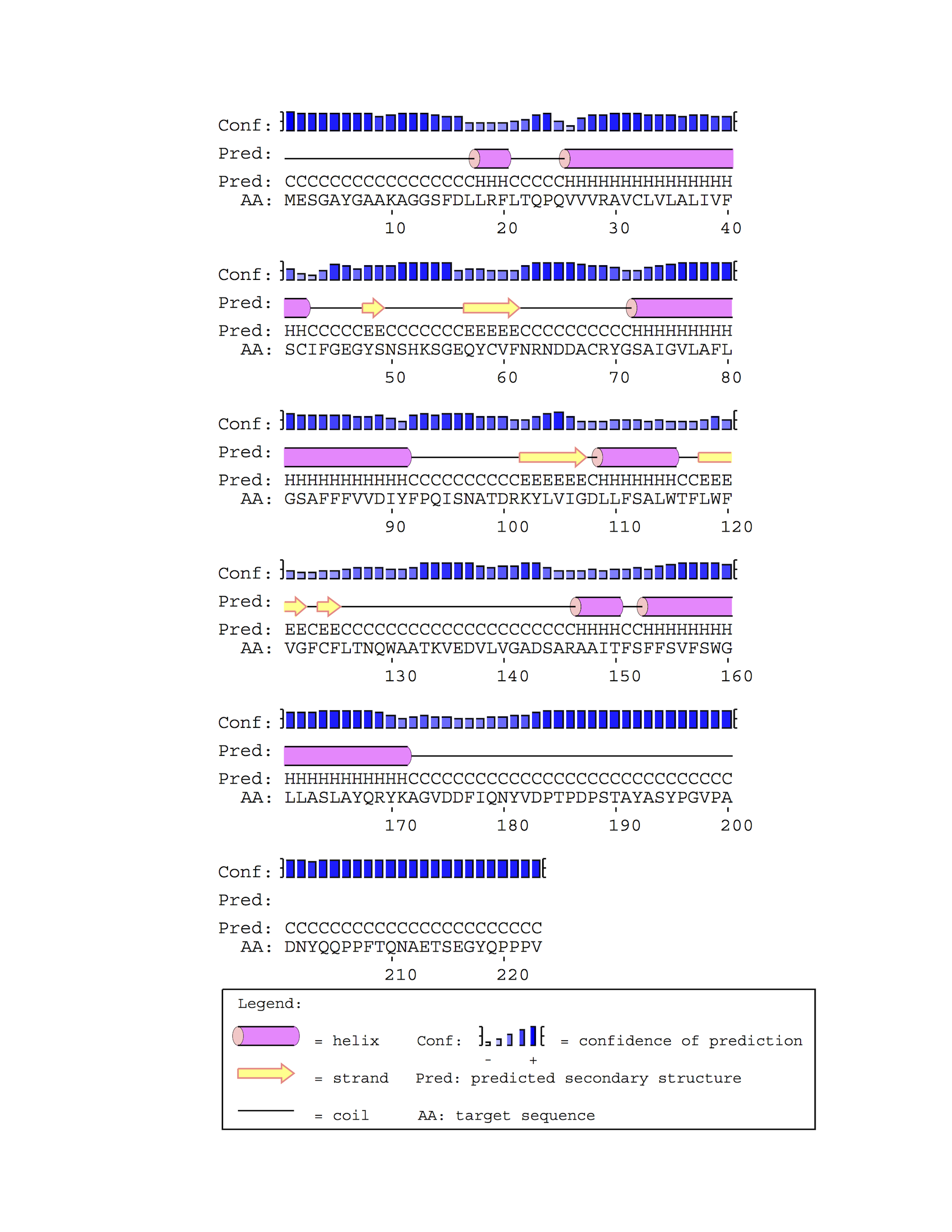

Supplement: S11 Fig — (TIFF) [file pgen.1007750.s011.tiff]
