# Supplementary material for: Synaptogyrin-2 influences replication of Porcine circovirus 2
Source: PLoS Genet. 2018 Oct 31;14(10):e1007750. doi: 10.1371/journal.pgen.1007750 (PMC6245838; doi:10.1371/journal.pgen.1007750)
Supplement: S2 Table — (DOCX) [file pgen.1007750.s013.docx]

Table SX. Haplotype frequency and substitution effect of the haplotypes located on a SSC12 LD block between *ALGA0110477* and *SYNGR2* and based on 16 DNA polymorphisms profiled in the PCV2b resource population.

| Haplotype | Frequency | Haplotype substitution effects | | | |
| --- | --- | --- | --- | --- | --- |
|  |  | Value | Std error | t Ratio | Prob>\|t\| |
| 1 | 0.32 | -12.5 | 1.997 | -6.28 | 2e^-9^ |
| 2 | 0.20 | 6.583 | 2.554 | 2.577 | 0.011 |
| 3 | 0.18 | 4.9 | 3.511 | 1.396 | 0.165 |
| 4 | 0.13 | 1.63 | 4.015 | 0.406 | 0.685 |
| 5 | 0.07 | 12.69 | 4.697 | 2.702 | 0.008 |
| 6 | 0.03 | 8.742 | 7.419 | 1.178 | 0.24 |
| 7 | 0.02 | -1.02 | 8.136 | -0.13 | 0.9 |
| 8 | 0.03 | 9.731 | 7.597 | 1.281 | 0.202 |
| 9 | 0.02 | 9.124 | 8.94 | 1.021 | 0.309 |
